# Kinetics of Myeloid Dendritic Cell Trafficking and Activation: Impact on Progressive, Nonprogressive and Controlled SIV Infections

**DOI:** 10.1371/journal.ppat.1003600

**Published:** 2013-10-03

**Authors:** Viskam Wijewardana, Jan Kristoff, Cuiling Xu, Dongzhu Ma, George Haret-Richter, Jennifer L. Stock, Benjamin B. Policicchio, Adam D. Mobley, Rebecca Nusbaum, Hadega Aamer, Anita Trichel, Ruy M. Ribeiro, Cristian Apetrei, Ivona Pandrea

**Affiliations:** 1 Center for Vaccine Research, School of Medicine, University of Pittsburgh, Pittsburgh, Pennsylvania, United States of America; 2 Department of Microbiology and Molecular Genetics, School of Medicine, University of Pittsburgh, Pittsburgh, Pennsylvania, United States of America; 3 Department of Pathology, School of Medicine, University of Pittsburgh, Pittsburgh, Pennsylvania, United States of America; 4 Department of Infectious Diseases and Microbiology, Graduate School of Public Health, University of Pittsburgh, Pittsburgh, Pennsylvania, United States of America; 5 Department of Biochemistry and Molecular Biology, Pennsylvania State University, University Park, Pennsylvania, United States of America; 6 Theoretical Biology and Biophysics Group, Los Alamos National Laboratory, Los Alamos, New Mexico, United States of America; National Institute of Allergy and Infectious Diseases, National Institutes of Health, United States of America

## Abstract

We assessed the role of myeloid dendritic cells (mDCs) in the outcome of SIV infection by comparing and contrasting their frequency, mobilization, phenotype, cytokine production and apoptosis in pathogenic (pigtailed macaques, PTMs), nonpathogenic (African green monkeys, AGMs) and controlled (rhesus macaques, RMs) SIVagmSab infection. Through the identification of recently replicating cells, we demonstrated that mDC mobilization from the bone marrow occurred in all species postinfection, being most prominent in RMs. Circulating mDCs were depleted with disease progression in PTMs, recovered to baseline values after the viral peak in AGMs, and significantly increased at the time of virus control in RMs. Rapid disease progression in PTMs was associated with low baseline levels and incomplete recovery of circulating mDCs during chronic infection. mDC recruitment to the intestine occurred in all pathogenic scenarios, but loss of mucosal mDCs was associated only with progressive infection. Sustained mDC immune activation occurred throughout infection in PTMs and was associated with increased bystander apoptosis in blood and intestine. Conversely, mDC activation occurred only during acute infection in nonprogressive and controlled infections. Postinfection, circulating mDCs rapidly became unresponsive to TLR7/8 stimulation in all species. Yet, stimulation with LPS, a bacterial product translocated in circulation only in SIV-infected PTMs, induced mDC hyperactivation, apoptosis and excessive production of proinflammatory cytokines. After infection, spontaneous production of proinflammatory cytokines by mucosal mDCs increased only in progressor PTMs. We thus propose that mDCs promote tolerance to SIV in the biological systems that lack intestinal dysfunction. In progressive infections, mDC loss and excessive activation of residual mDCs by SIV and additional stimuli, such as translocated microbial products, enhance generalized immune activation and inflammation. Our results thus provide a mechanistic basis for the role of mDCs in the pathogenesis of AIDS and elucidate the causes of mDC loss during progressive HIV/SIV infections.

## Author Summary

Myeloid dendritic cells (mDCs) are potent antigen-presenting cells that regulate both innate and adaptive immune responses and act as “watch-dogs”, sensing and controlling aberrant immune activation; as such, they may significantly impact the outcome of HIV/SIV infection. By comparing and contrasting the frequency, function, migration to tissues and levels of activation and apoptosis in progressive, nonprogressive and elite-controlled SIV infections, we investigated the mechanisms responsible for mDC loss in HIV/SIV infection and their role in driving progression to AIDS. We report that progression to AIDS is associated with low mDC preinfection levels and depletion throughout infection, due to massive migration of these cells to mucosal sites and excessive cell death by apoptosis. We also show that residual mDCs from blood and intestine have a high capacity to produce proinflammatory cytokines, thus contributing to the increased immune activation and inflammation characteristic of progressive infections.

## Introduction

Myeloid dendritic cells (mDCs) are potent antigen-presenting cells which are responsible for initiating both innate and adaptive immune responses. mDCs stimulate NK, B and T cells [1], [2], but can also act as “watch-dogs” to sense and regulate aberrant immune activation, induce tolerance and thus prevent autoimmune diseases [2]. The ability of mDCs to act as immune sentinels is conferred by their capacity to migrate into lymphoid organs where they secrete cytokines and initiate immune responses [2]. Altogether these characteristics suggest that mDCs may have a critical role in the pathogenesis of human and simian immunodeficiency virus (HIV/SIV) infections, in which progression to AIDS is driven by excessive generalized immune activation and inflammation [3].

A considerable amount of correlative data reporting changes in mDC counts during pathogenic HIV/SIVmac infections has been published [4]–[14], showing that mDC depletion from circulation occurs during acute HIV/SIV infection, at the time of peak viremia, and persists throughout the chronic infection and progression to AIDS [4], [7], [11], [14]. The exact mechanism of mDC depletion is not yet understood. In SIV-infected nonhuman primates (NHPs), mDC loss is inversely correlated with virus loads (VLs) [7], the NHPs that rapidly progress to AIDS having high VLs and a profound loss of mDCs [3]. Conversely, SIVmac-infected rhesus macaques (RMs) with normal or delayed disease progression have lower VLs and increased numbers of circulating mDCs [7]. NHP studies corroborate reports in HIV-infected patients showing that antiretroviral therapy restores mDCs and suggesting that direct killing of mDCs by the virus is a potential mechanism. mDCs can indeed be infected by HIV-1, the virus burden in mDCs isolated from chronically HIV-1-infected patients being considerable [15]. However, mDC depletion from circulation was also reported for HIV-2–infected patients and RMs infected with SIVmac (which is closely related to HIV-2), albeit mDCs appear to be less susceptible to HIV-2 infection [16], suggesting that additional factors may be responsible for mDC depletion. Other studies have suggested that mDC loss from the circulation may occur through either increased apoptosis [17] or mDC recruitment to the lymph nodes (LNs) [7], [18]–[20].

While mDCs are pivotal in shaping the mucosal microenvironment, no study to date has been carried out in either humans or animal models of HIV infection to explore whether or not the loss of circulating mDCs is due to recruitment to mucosal tissues such as the intestine, which is the main target of viral replication and where a high degree of inflammation occurs [21]. It was previously shown that a loss of the chemokine balance in the LN environment occurs in SIV-infected macaques and results in recruitment of mDCs to LNs [7], . Similar changes may also occur in the mucosal tissues, leading to recruitment of immune cells, including activated mDCs, to these sites [24].

Thus, the exact role of mDCs in the pathogenesis of AIDS is unknown, yet may be essential for designing better vaccines and therapeutic interventions. Loss of mDCs during pathogenic HIV/SIV infections prevents a clear understanding of their precise role in HIV/SIV pathogenesis. One option towards realizing this goal may rely on comparing and contrasting the dynamics, trafficking and function of mDCs in lentiviral infections with variable pathogenic outcomes.

There are three pathogenic outcomes of lentiviral infections: (a) The *persistent progressive infection* occurs in the majority of cases of HIV infection and SIVmac/smm infection of macaque species and is characterized by (i) massive, continuous viral replication [25]–[27], with VL setpoint being predictive for the time of progression to AIDS [28]–[30]; (ii) continuous depletion of CD4^+^ T cells from the peripheral blood [31], [32] that is more pronounced at mucosal sites [21], [33]–[35]; and (iii) high levels of T cell immune activation [36], [37], the magnitude of which has been reported to be predictive of disease progression [36], [37]. The interaction between these factors cripples the immune system and eventually results in severe immunodeficiency and death [31], [32], [38]. (b) The *persistent nonprogressive infection* is observed in African NHPs that are natural hosts of SIV, such as African green monkeys (AGMs), sooty mangabeys (SMs) and mandrills (reviewed in [39]), and is characterized by (i) active viral replication, with setpoint VLs similar to or even higher than those reported in pathogenic infections [40], [41]; (ii) transient depletion of peripheral CD4^+^ T cells during acute infection that rebound to preinfection levels during chronic infection [39], [40]; (iii) significant CD4^+^ T cell depletion in the intestine that can be partially restored during the chronic infection, regardless of significant viral replication [41]–[43]; (iv) low levels of CD4^+^ T cells expressing the CCR5 coreceptor in blood and tissues [44], [45], and (v) transient and moderate increases in immune activation and T cell proliferation during acute infection, which is resolved to near baseline levels with the transition to chronic infection [42], [46], [47]. Altogether, the action of these factors results in an active, persistent SIV infection, which only rarely progresses to AIDS in natural hosts [48]. (c) The *controlled infection* occurs in a minority (1–5%) of HIV-infected individuals, which are defined as long-term nonprogressors (LTNPs). LTNPs that have undetectable VLs for at least 1 year are referred to as elite controllers (ECs) (reviewed in [49]). The main characteristics of LTNP infections are (i) infection for more than 7 years; (ii) stable CD4^+^ T cell counts greater than 600 cells/µl; (iii) low/undetectable levels of HIV in the peripheral blood; (iv) no symptoms of HIV-induced disease; and (v) presence of a vigorous immune response against HIV, with multifunctional, persistent CD4 and CD8 responses. ECs that control immune activation in addition to VLs are referred to as superelite controllers. Similarly, SIV infection in NHPs may result in a controlled infection: in a fraction of Indian RMs infected with SIVmac and carrying restrictive MHC genotypes (MaMu A*01, B*17, B*08), in Chinese macaques, or upon cross-species transmission of SIVs [50]–[55]. By exposing RMs to SIVagmSab, we have recently developed an animal model in which superelite control/functional cure of SIV infection occurs in 100% of cases [56].

To date, all information on mDCs in HIV/SIV infections is derived from studies performed on persistent progressive (pathogenic) infections (reviewed in [15]). Very few of these studies focused on mDCs at mucosal sites [57]–[60]. Furthermore, to our knowledge, the role of mDCs in the pathogenesis of either persistent nonprogressive or controlled lentiviral infections has not yet been established.

Hence, to test the hypothesis that distinct mDC profiles are associated with particular pathogenic outcomes, we dissected the kinetics of mDCs in blood, LNs and at mucosal sites in persistent progressive infections in PTMs, nonprogressive persistent infections in AGMs and controlled infections in RMs after inoculation with the same virus strain (SIVagmSab). We report that the three pathological outcomes are associated with different dynamics of mDCs. To explain these outcomes, we examined the mobilization, migration, activation and apoptosis of mDCs in three animal models. We corroborated this descriptive data with mDC functional studies using cells isolated from blood and intestine from SIVagmSab-infected PTMs, AGMs and RMs. Altogether, our results indicate that mDCs play a significant role in driving the outcome of SIV infection.

## Results

### Pathogenic diversity of SIVagm infection in PTMs, AGMs and RMs

Five PTMs, four AGMs, and five RMs were intravenously infected with the 300 TCID50 of SIVagmSab92018 [61]. All of the PTMs developed significant lymphadenopathy during the acute infection and exhibited signs of disease progression during the follow-up: two experienced weight loss during acute infection, followed by rapid progression to AIDS (78 and 104 days postinfection, dpi); two additional PTMs progressed to AIDS within a year postinfection; the remaining PTM did not progress to AIDS during the follow-up, but showed profound CD4^+^ T cell depletion and persistently high VLs, which are indicative of progressive, pathogenic infection. The PTMs with AIDS presented with anorexia/cachexia, diarrhea, LN and spleen atrophy, neurological disease, thrombotic microangiopathy (TMA), glomerulonephropathy and myocarditis. RMs had marked lymphadenopathy during acute infection. No clinical sign of infection was observed during acute infection in AGMs or during chronic infection in AGMs and RMs.

**Figure 1:**
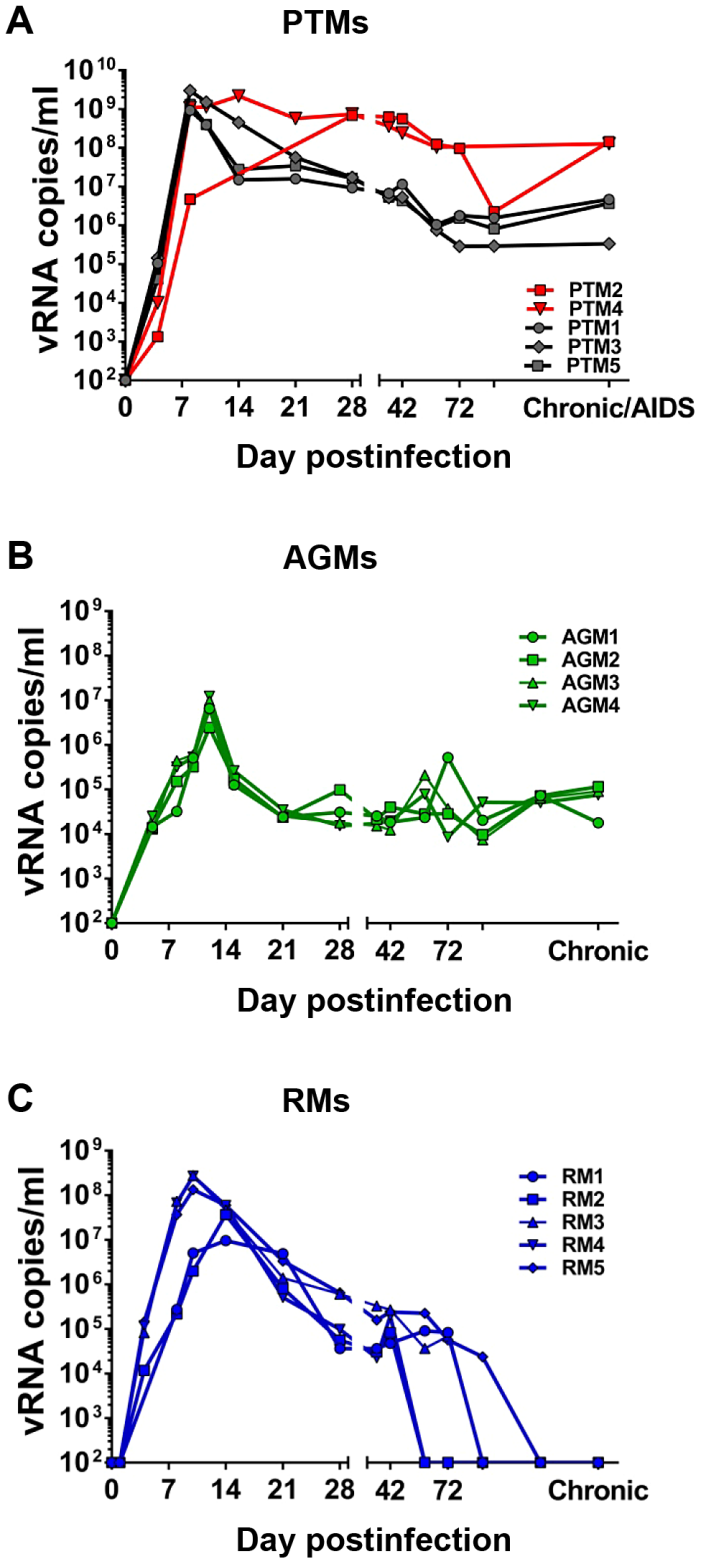
SIVagm plasma viral loads in pathogenic, persistent nonprogressive and elite-controlled infections. (a) SIVagm-infected pigtailed macaques (PTMs), with rapid progressors depicted in red dots and lines and normal progressors depicted in black dots and lines, (b) SIVagm-infected African green monkeys (AGMs-green) and (c) SIVagm-infected rhesus macaques (RMs-blue).

Acute VLs peaked at day 8–14 dpi (Figure 1a, b and c) and were similarly high in all three species. However, there were slight species-specific variations, with PTMs having the highest VLs, followed by RMs and AGMs; the different peak levels correlated with different levels of CD4^+^ T cells expressing the CCR5 molecule (the target cells of SIVagmSab), which are the highest in PTMs, intermediate in RMs and the lowest in AGMs [44]. In stark contrast to this uniform viral replication during the acute infection, chronic viral replication patterns were completely divergent between the three models (Figure 1): VLs remained high in PTMs and showed two patterns of chronic replication: noncontrolled in the two rapid progressors (chronic VLs of 10^8^ SIVagmSab RNA copies/ml, i.e., a reduction of less than 1.5 logs from the peak VLs); in the remaining three PTMs, a relative control was observed (chronic VLs of 10^5^–10^6^ SIVagmSab RNA copies/ml, i.e., a contraction of 3 logs from the peak VLs) (Figure 1a). Conversely, in AGMs VLs reached a setpoint level of 4×10^4^ copies/ml by 42 dpi, which was maintained throughout the follow-up (Figure 1b), in agreement with our previous studies showing that this setpoint can be maintained for decades in AGMs [62]. Finally, SIVagmSab-infected RMs gradually controlled VLs, which became undetectable from 60 dpi on and remained so throughout the follow-up (Figure 1c), in agreement with our previous studies showing that viral control can extend beyond 5–6 years [56].

### PTMs with lower circulating mDC counts prior to infection experience rapid progression to AIDS

**Figure 2:**
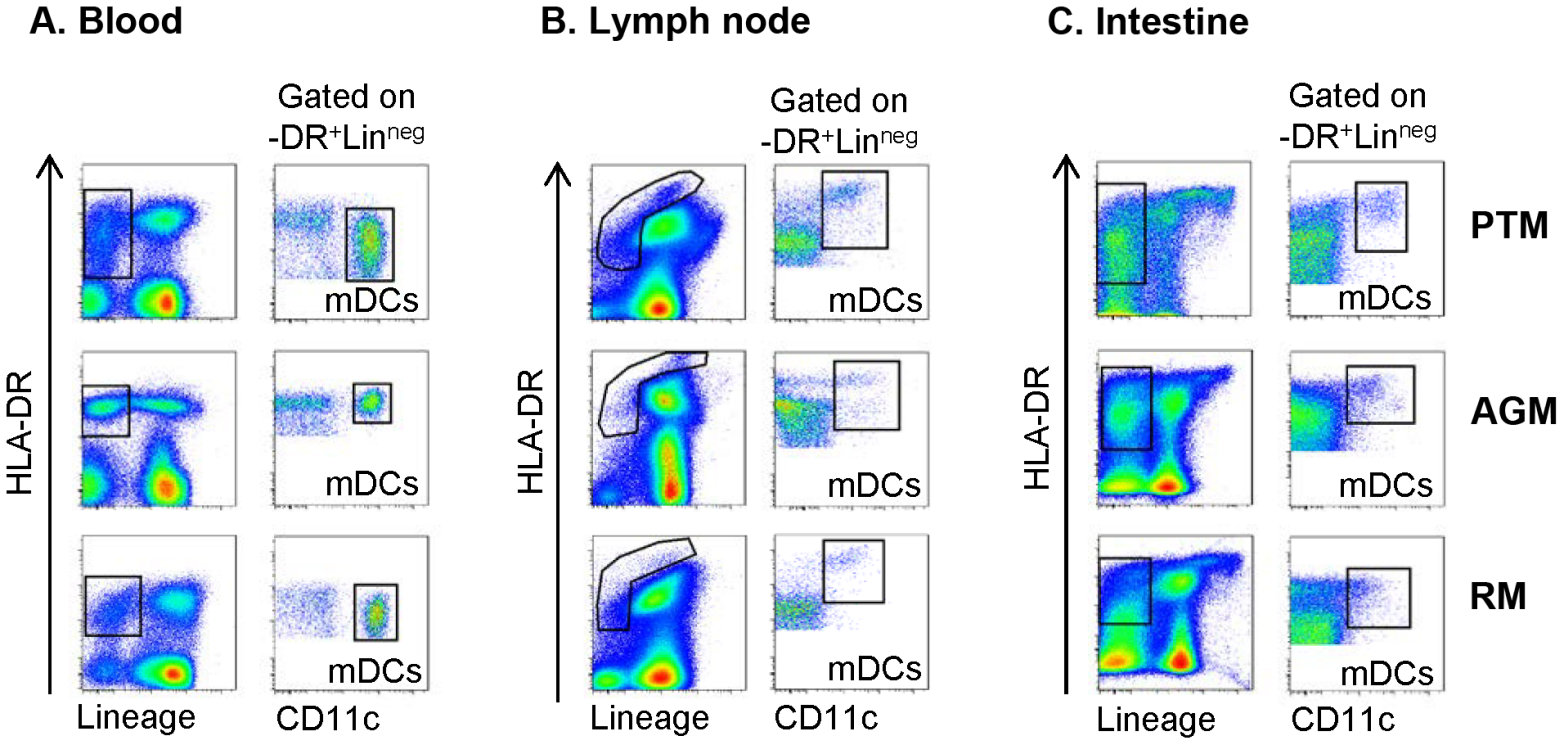
Phenotypic characterization of mDCs in blood, lymph nodes (LNs) and intestine of pigtailed macaques (PTMs), African green monkeys (AGMs) and rhesus macaques (RMs). Representative flow cytometry plots illustrating the gating strategy used to define CD11c^+^ mDC within the Lineage^neg^ (Lin^−^) HLA-DR^+^ fraction of (a) blood, (b) peripheral LN cell suspensions, and (c) intestinal cell suspensions. Presented plots are generated from samples collected prior to SIVagmSab infection. The same gating strategy was used throughout the study.

As a first step of dissecting the role of mDCs in driving different outcomes of SIV infection, we assessed their characteristics in blood, LN and intestine in the three species of NHPs prior to infection. We first characterized mDCs in PTMs, AGMs and RMs by applying the strategy previously reported to identify mDCs in RMs [6], [7] and showed that selection based on Lineage^neg^, HLA-DR^+^ and CD11c^+^ identified mDCs with similar phenotypic characteristics in all three species in blood (Figure 2a), LNs (Figure 2b) and intestine (Figure 2c). Characterization of mDCs from blood, LNs and intestinal mucosa revealed, however, different levels of HLA-DR expression between the three sites (Figure 2): low in peripheral blood, high in the LN and intestine. This phenotypic variation is probably due to differences in mDC maturation stages: immature mDCs with low HLA-DR expression are present in circulation, while more mature mDCs expressing higher levels of HLA-DR are found in the LNs and mucosal tissues.

Comparison of mDC baseline levels between PTMs showed that the two rapid progressor PTMs had lower peripheral baseline mDC counts compared to the normal progressors. As the number of rapid progressor PTMs included in the study group precluded statistical analysis of the data, we compared the baseline levels of mDCs between rapid and normal progressors in a larger cohort of PTMs from previous studies. This analysis confirmed an inverse correlation between the low numbers of circulating mDCs prior to infection and the rapid disease progression of SIVagmSab-infected PTMs (Figure S1), thus suggesting a protective role of mDCs during the course of HIV/SIV infection.

### Circulating mDCs are depleted throughout pathogenic infection (PTMs), transiently depleted in nonpathogenic infection (AGMs), and not depleted in controlled infections (RMs)

**Figure 3:**
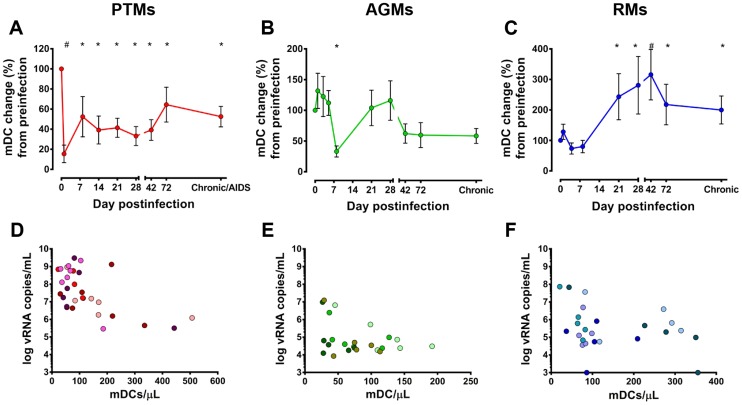
Distinct dynamics of circulating mDCs in progressive (pigtailed macaques-PTMs), nonprogressive (African green monkeys-AGMs) and controlled (rhesus macaques-RMs) SIVagm infections. mDC counts are illustrated as a percentage of the baseline mDC counts recorded prior to SIVagmSab infection. (a) PTMs; (b) AGMs; and (c) RM. * signifies *p*<0.05; # signifies *p*<0.01 changes from the baseline levels within the same animal group (Anova). Correlation between the absolute counts of circulating mDCs and plasma viral loads (data collected between 8 and 120 dpi). (d) PTMs; (e) AGMs; and (f) RMs. Each animal is depicted in a specific color.

Using a random effects model to analyze the dynamics of mDC during infection in all animals, we found that there were significant differences in the changes over time across the 3 infection models (p = 0.0003). We then analyzed in more detail the changes in circulating mDCs at key time points of SIVagm infection in the three species to determine whether or not differences in the dynamics of peripheral mDCs could explain the different pathogenic outcomes. Although dynamics of acute virus replication were similar in all three infections, the number of mDCs significantly dropped only in PTMs (Figure 3a) and AGMs (Figure 3b), but not in RMs (Figure 3c). Furthermore, the duration and timing of acute mDC depletion were different in PTMs and AGMs: rapid (from 1 dpi) and maintained throughout the acute infection in PTMs (Figure 3a), while very transient, reaching significance only around the peak of viral replication in AGMs (Figure 3b). mDC depletion from circulation prior to detectable viremia is not surprising, as in the mouse model the virus is already present in the LNs and that DCs are migrating to the LNs carrying the virus 30 minutes post intravenous HIV inoculation [63].

Major differences in the dynamics of mDCs became discernible between the three models during chronic SIV infection. In PTMs, progression to AIDS was characterized by a significant decline of mDCs (Figures 3a). To confirm that mDC depletion is specifically associated with disease progression, we compared mDC counts prior to SIV infection and during chronic SIV infection in animals included in other studies and showed that mDCs are indeed lost with disease progression (Figure S2). Our results thus corroborate previous reports documenting loss of peripheral mDCs during pathogenic HIV/SIV infections [7].

In AGMs, mDC levels returned to near baseline levels with the transition to chronic infection, then were maintained throughout follow-up (Figure 3b).

Finally, in RMs, a 300% increase from the baseline mDC counts occurred during early chronic infection, coincident with the control of viral replication (Figure 3c). Once the VLs were controlled, the circulating mDCs declined in RMs but remained significantly increased compared to baseline values long after the viremia was completely controlled (Figure 3c).

Our results show that the degree of mDC recovery during chronic infection rather than their acute depletion predicts disease progression in SIV-infected NHPs. These results corroborate previous studies showing that higher peripheral mDC counts are associated with slower disease progression in SIVmac-infected RMs [7].

### Depletion of peripheral mDCs is not due to direct virus killing

We further investigated the mechanisms responsible for the distinct dynamics of peripheral mDCs observed in progressors *vs* nonprogressors *vs* controllers.

To determine if mDC depletion is due to direct virus killing, we first correlated the absolute mDC counts with the VLs in each species between 8 and 120 dpi. The use of the same virus to infect these three distinct NHP species eliminates the variables related to the ability of different SIV/HIV to infect and thus directly kill mDCs. A strong negative correlation (using mixed-effects models) was found in AGMs between higher VLs and lower mDC counts (p = 0.006) (Figure 3e), yet we could not establish any significant association of the same parameters in PTMs (p = 0.43) (Figure 3d) and RMs (p = 0.15) (Figure 3f) infected with SIVagm.

To assess the role of virus killing in inducing mDC loss, we further sorted mDCs from the LNs collected from all three species and showed that mDCs from PTMs, AGMs and RMs do not harbor detectable levels of SIVagmSab DNA (data not shown). Conversely, a cellular fraction containing Lineage^+^ cells (and representing a mixture of CD3^+^ T cells and B lymphocytes) harbored high amounts of virus in all the three species included (data not shown). This result is not surprising, as the recent description of SAMHD-1 restriction factor suggested that dendritic cells cannot be infected by primate lentiviruses that do not contain a *vpx* gene, as is the case of SIVagmSab92018 [64].

### Peripheral mDC loss is not due to lack of mobilization of these cells from the bone marrow

We next investigated if differences in mDC mobilization from the bone marrow in SIVagmSab-infected PTMs, AGMs and RMs can explain the variation in numbers of circulating mDCs in these three species.

Since mDCs rarely undergo division in the periphery and tissues [2] and Ki-67 antigen is not expressed by cells in the resting phase (G_0_), Ki-67 expression by circulating mDCs is indicative of active or recent cell division, i.e., production and mobilization from bone marrow, the primary site where mDCs undergo cell division and differentiation [65]. Overall, there was a significant difference in the dynamics of Ki67^+^ mDC in circulation among the three species (p = 0.0054).

**Figure 4:**
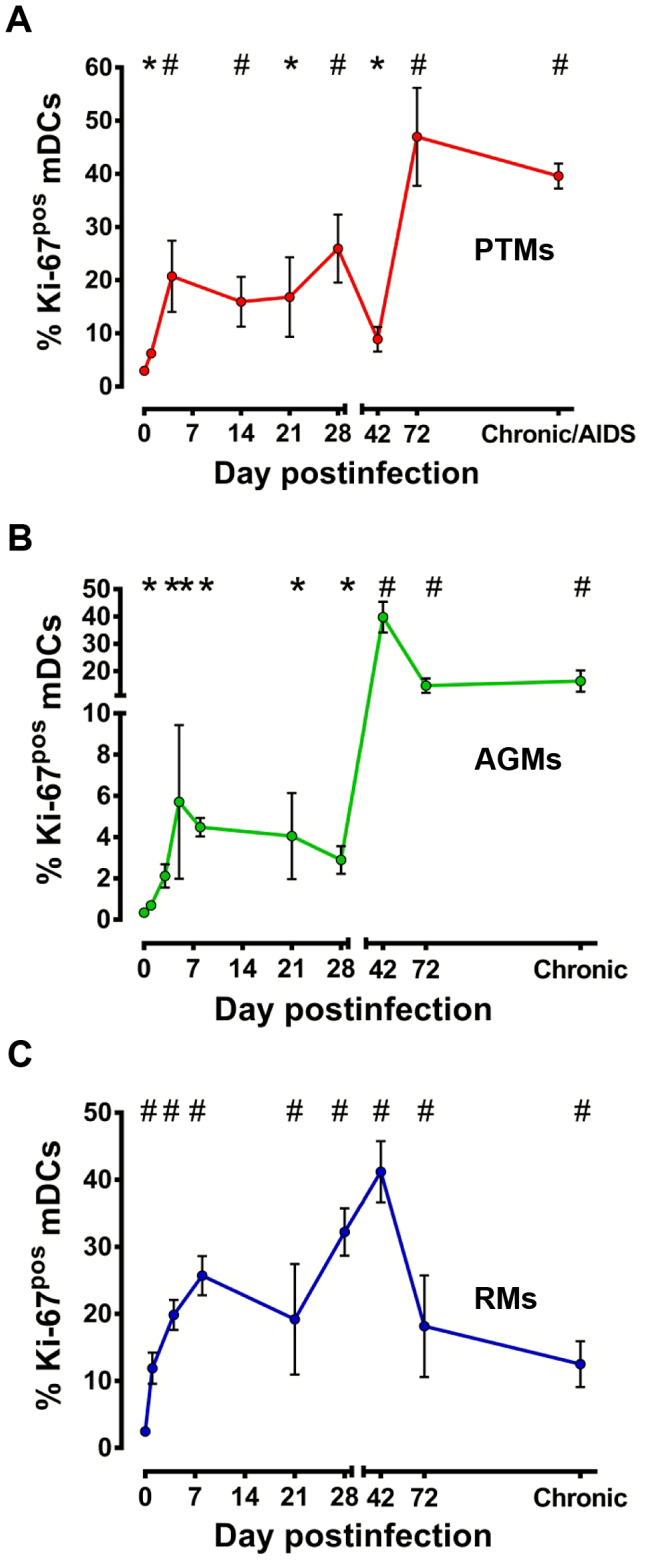
mDC mobilization from bone marrow to periphery occurs in pathogenic (PTMs), persistent nonprogressive (AGMs) and elite-controlled (RMs) SIVagm infections. Assessment of Ki-67 levels on circulating mDCs in SIVagmSab-infected (a) PTMs; (b) AGMs; and (c) RMs. * signifies *p*<0.05; # signifies *p*<0.01 changes from the baseline levels within the same animal group (Anova).

Ki-67 expression by peripheral mDCs increased in the PTMs throughout SIVagmSab infection (Figure 4a), thus excluding the possibility that loss of mDC in this species is due to their lack of mobilization from the bone marrow. Even rapid progressor PTMs that had lower mDC counts at baseline showed a clear tendency to restore these cells in the periphery through increased mDC mobilization from bone marrow (Figure S3). This shows that the massive mDC loss is not due to a defect of bone marrow specific to the rapid progressors, but rather to other factors.

Increased mDC mobilization from BM was also observed in AGMs (Figure 4b) and RMs (Figure 4C) in the early stages of SIVagm infection and was maintained throughout the follow-up. Interestingly, the highest mDC mobilization from bone marrow was observed in the RM controllers (Figure 4C), which may be the mechanism behind the increased mDC counts observed in SIVagm-infected RMs.

However, with the exception chronic RM infection, in which significant increases in Ki-67 expression by mDC are accompanied by significant increases in mDC counts (Figure 3c), there were no other direct positive correlation between the degree of mDC mobilization from bone marrow and the peripheral mDC counts in the other species.

Our results indicate that mDC mobilization from bone marrow occurs very early postinfection in all pathogenic scenarios and is maintained unaltered even during progression to AIDS. Yet, this increased mDC mobilization alone is not sufficient to preserve healthy counts of peripheral mDC in SIV-infected NHPs, and most likely other factors play a significant accessory role in the preservation or loss of these cells from the circulation.

### Increased mDC frequency in the intestine is associated with nonprogressive and controlled SIV infections

**Figure 5:**
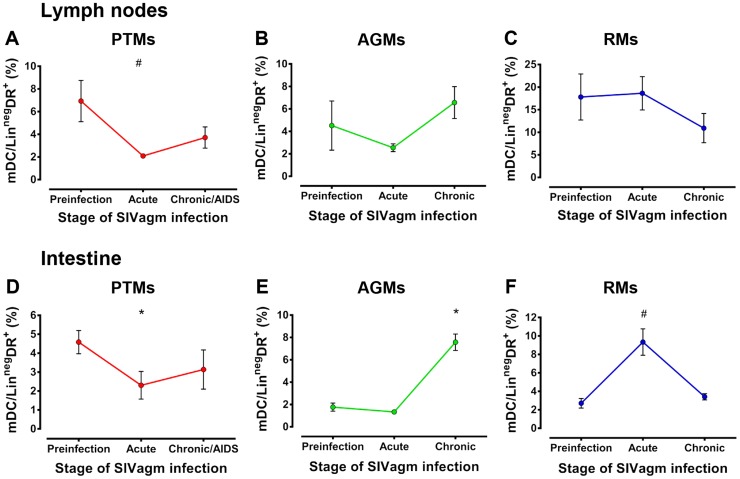
Increases of mDCs at mucosal sites are associated with nonprogressive infection. Dynamics of mDCs are shown in the lymph nodes in (a) PTMs, (b) AGMs and (c) RMs and in the intestine in (d) PTMs, (e) AGMs and (f) RMs. * signifies *p*<0.05; # signifies *p*<0.01 changes from the baseline levels within the same animal group (Anova).

To determine whether or not mDC recruitment to LNs and intestine is responsible for their loss from circulation and to establish if this outcome drives disease progression, we first used flow cytometry to assess mDC dynamics in the LNs and intestine during acute and chronic SIV infection in the three types of SIV infection. We found differences in the changes in mDC frequency in LN over time across the species (p = 0.0162), as well as trends toward a difference in the intestine (p = 0.0645). Assessment of the mDC frequency in the LNs and intestine showed that they decreased during acute infection in PTMs in both compartments (Figure 5a and 5d) and were only partially restored during chronic infection. While mDC depletion in the intestine did not reach statistical significance during chronic infection of PTMs included in this study (Figure 5d) or in a larger cohort including PTMs from our previous studies (Figure S4a), a subset of mDCs expressing CD103^+^ were preferentially lost at the mucosal sites in chronically-infected PTMs (Figure S4b). This result corroborates data previously reported for other pathogenic SIV infections [60]. No significant changes in mDC counts occurred in the LNs in AGMs and RMs (Figures 5b and c), while mDC percentages were significantly increased in the intestine in these two species at different time points of SIVagmSab infection (Figures 5e and f).

These immunophenotypic results were confirmed by immunohistochemistry (IHC) for CD11c (Figures S5, S6, S7 and S8). In general, there was a good match between IHC and flow cytometry data, indicating acute mDC depletion in the LN (Figure S5 a–c) and intestine (Figure S6) in the progressive infection of the PTMs, increased during chronic infection in the gut of nonprogressor AGMs (Figure S7 d–i) and increased during acute infection in RM controllers (Figure S8 d–i).

IHC showed that, in the intestine, the majority of the CD11c^+^ cells were located in the Peyer's Patches (Figure S7 d–f) and in the lymphoid aggregates (Figure S8 d–f), with only a small fraction of mDCs being present in the lamina propria (Figures S6a–c, S7g–I and S8d–i). This points to potential differences in CD11c dynamics in different gut segments (i.e., inductive *vs* effector sites). Similarly, IHC results revealed differences in the dynamics of CD11c expression between superficial and mesenteric LNs in the pathogenic infection of PTMs (Figure S5).

Our data showing increases in the mDC population in the intestinal compartment in persistent nonprogressive SIVagmSab infection in AGMs and in controlled SIVagmSab infection in RMs, suggest that mDC recruitment to these sites is not deleterious for SIV/HIV pathogenesis. In contrast, loss of mDCs from mucosal sites was correlated with disease progression in SIVagmSab-infected PTMs.

### Analysis of additional mDC mobilization markers indicates mDC recruitment to LNs and intestine in response to pathogenic, nonpathogenic and controlled SIV infections

The decreased numbers of mDCs in the LNs and intestine of PTMs may result from a lack of recruitment to these tissues. Yet, increased apoptosis of mDCs from circulation and LNs was previously reported in progressive HIV/SIVmac infections [7]. Consequently, we could not rule out mDC loss from lymphoid and mucosal tissues through a similar mechanism, and we assessed additional markers of mDC migration and recruitment to lymphoid sites.

**Figure 6:**
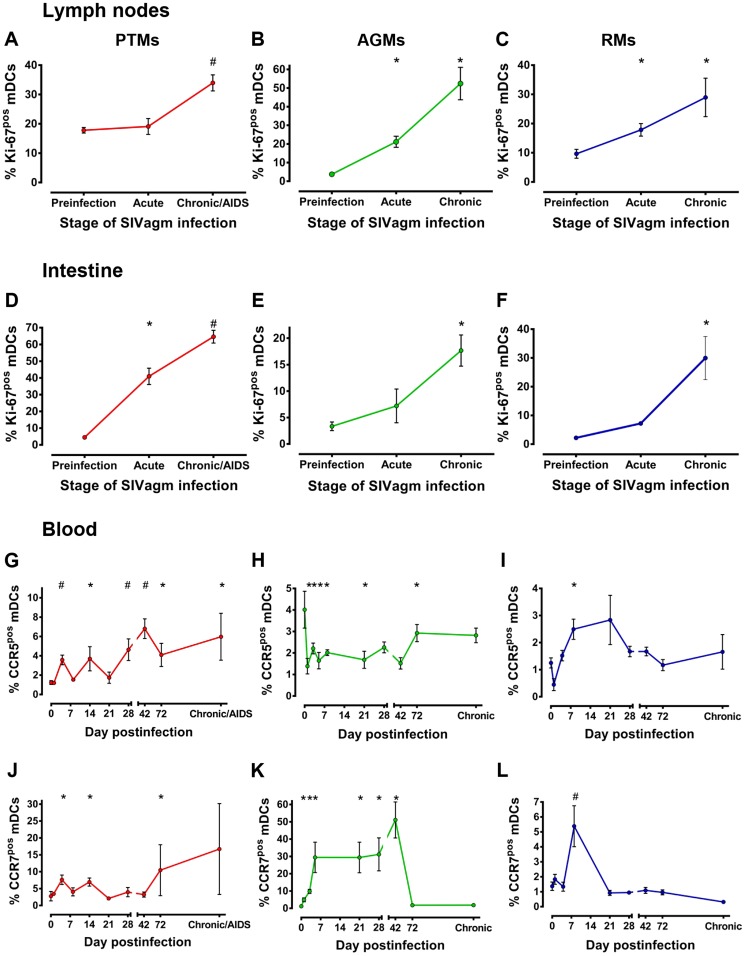
mDC mobilization to LNs and intestine occurs in pathogenic (PTMs), persistent nonprogressive (AGMs) and elite-controlled (RMs) SIVagmSab infections. Assessment of Ki-67 levels on mDCs isolated from lymph nodes: SIVagmSab-infected (a) PTMs, (b) AGMs and (c) RMs and intestine: SIVagmSab-infected (d) PTMs, (e) AGMs and (f) RMs. Assessment of CCR5 expression on circulating mDCs in SIVagmSab-infected (g) PTMs, (h) AGMs and (i) RMs. Assessment of CCR7 expression on circulating mDCs of (j) PTMs, (k) AGMs and (l) RMs. * signifies *p*<0.05; # signifies *p*<0.01 changes from the baseline levels within the same animal group (Anova).

We measured Ki-67 expression on mDCs isolated from LN and intestine as a marker of their recent mobilization at these sites. We found significant differences over time across the three species, both in LN (p = 0.0001) and in the intestine (p = 0.0319). Ki-67 expression rapidly increased on mDCs in the LNs and was maintained at high levels throughout infection in all three species, the highest increase being observed in AGMs (Figures 6a, b, c). Slight, but consistent increases of Ki-67 expression by mucosal mDCs were observed in AGMs and RMs (Figures 6e, f), especially during chronic SIV infection. Increased mDC mobilization to the intestine may explain the increased numbers of mDCs observed at this site in nonprogressive infections. Surprisingly, however, the most prominent increase in Ki-67 expression occurred in the intestine of SIVagmSab-infected PTMs, starting from the acute infection throughout the follow-up (Figure 6d), suggesting that mDCs are also recruited to mucosal site in progressive infections. This observation is also supported by the observation of significant increases in α4β7 integrin expression on circulating mDCs isolated from both AGMs and PTMs (Figure S9), confirming their recruitment to the gut in the progressive infection [24]. Low levels of mDCs in the gut despite their documented massive recruitment indicate that additional factors are responsible for mDC depletion in the intestine of SIVagmSab-infected PTMs.

To complete the characterization of mDC migration to either LNs or mucosal tissues, we examined the expression of chemokine receptors on circulating mDCs. We reasoned that, if mDC recruitment to LNs and tissues is driven by specific chemokine signaling, circulating mDCs that migrated to certain effector sites should express the necessary chemokine receptors to respond to chemokines from tissues or LNs. We thus measured the levels of CCR7, a receptor for two chemokines (CCL19 and CCL 21) usually expressed in the LNs, [66]–[68] and CCR5, a receptor for chemokines (CCL3, CCL4 and CCL5) usually elevated in inflamed mucosal tissues [69], to assess mDC migration to either LNs or mucosal tissues.

In pathogenic SIV infection of PTMs, expression of both CCR5 and CCR7 chemokine receptors increased on peripheral mDCs, with a more prominent increase occurring for CCR5 (Figures 6g and j).

In the nonpathogenic SIV infection of AGMs, very high CCR7 expression, but lower CCR5 expression was observed in the peripheral mDCs (Figures 6h and k). The decreased CCR5 expression on circulating mDCs from SIVagmSab-infected AGMs may suggest their lower recruitment to intestine in AGMs *vs* PTMs. However, the increases observed for all the other mobilization/recruitment markers and the observation that mDCs are increased in the intestine support recruitment of mDCs to the gut in AGMs. Finally, in the controlled SIVagmSab infection of RMs, circulating mDCs showed transient but significant increases of both CCR7 and CCR5 expression (Figure 6i and l).

Altogether, these results strongly support recruitment of mDCs to LNs and intestine in all the pathogenic scenarios.

### mDCs undergo apoptosis in blood and intestine in pathogenic but not in nonpathogenic or controlled SIV infections

**Figure 7:**
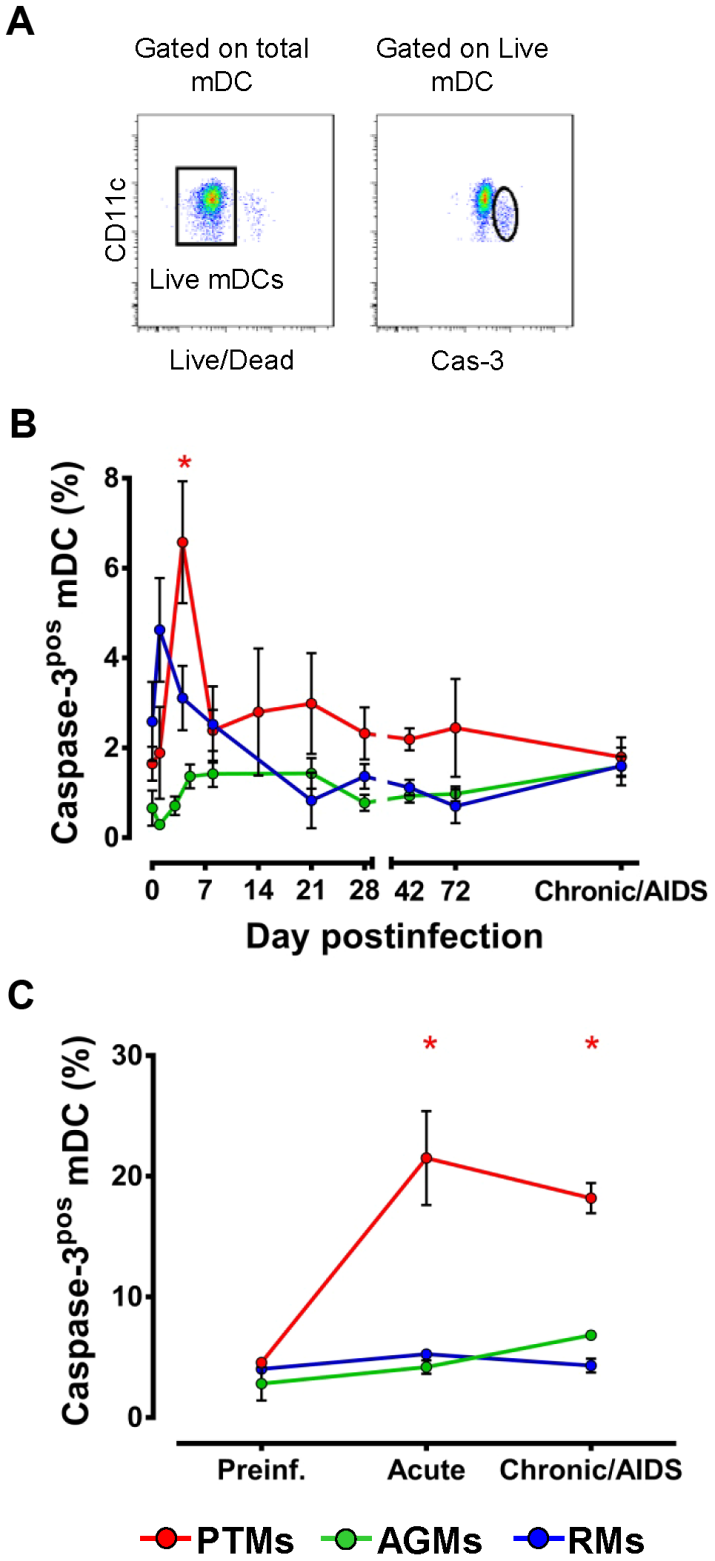
mDCs undergo increased apoptosis both in blood and intestine during pathogenic SIVagmSab infection. (a) Representative flow cytometry plots illustrating the gating strategy used to identify mDCs expressing activated caspase-3 (Cas-3). (b) Dynamics of activated Caspase-3 expression on circulating mDCs isolated from blood in PTMs (red), AGMs (green) and RMs (blue). (c) Dynamics of activated Caspase-3 on mDCs isolated from intestine in PTMs (red), AGMs (green) and RMs (blue). Shown are averages+SEM. * signifies *p*<0.05 within groups relative to preinfection.

Two lines of evidence suggest that significant mDC apoptosis occurs in the pathogenic SIV infection of PTMs: (a) increased mDC mobilization from bone marrow (increased Ki-67), yet decreased levels of circulating mDCs; and (b) migration of activated mDCs to the intestine (increased CCR5 and α4β7 expression on circulating mDCs and increased Ki-67 expression on intestinal mDCs), without consistent increase of mDCs in the intestine. As a result, we hypothesized that while mDCs are mobilized to mucosal tissues, once they arrive at this particular compartment they undergo apoptosis, which offsets their influx. To test this hypothesis, we examined the expression of activated Caspase-3 by mDCs from both intestine and peripheral blood. We gated on live mDCs that expressed activated Caspase-3 to specifically identify the cells that undergo apoptosis (Figure 7a). Circulating mDCs from PTMs expressed increased levels of activated Caspase-3 during SIV infection (Figure 7b). Conversely, activated Caspase-3 expression of mDCs did not significantly change at any time during SIV infection in AGMs or RMs (Figure 7b). Similar kinetics of apoptotic mDCs were observed in the intestine, with activated Caspase-3 expression being increased during acute and chronic infection in PTMs, while remaining unchanged throughout infection in AGMs and RMs (Figure 7c). In conclusion, our data clearly show that mDC loss in progressive infections results through bystander apoptosis, which is particularly severe at mucosal sites.

### mDC immune activation is increased throughout progressive infection and only transiently elevated during nonprogressive and controlled infections

Our further goal was to determine if mDC loss observed during progressive SIV infection in PTMs is due to immune activation-induced bystander apoptosis. We therefore assessed expression of CD80 and CD95 receptors on mDCs isolated from blood and tissues from PTMs, AGMs and RMs. Both CD80 and CD95 have been reported to increase during activation of mDCs [70], [71]. Meanwhile, mDCs that express CD95 can potentially undergo apoptosis [72].

**Figure 8:**
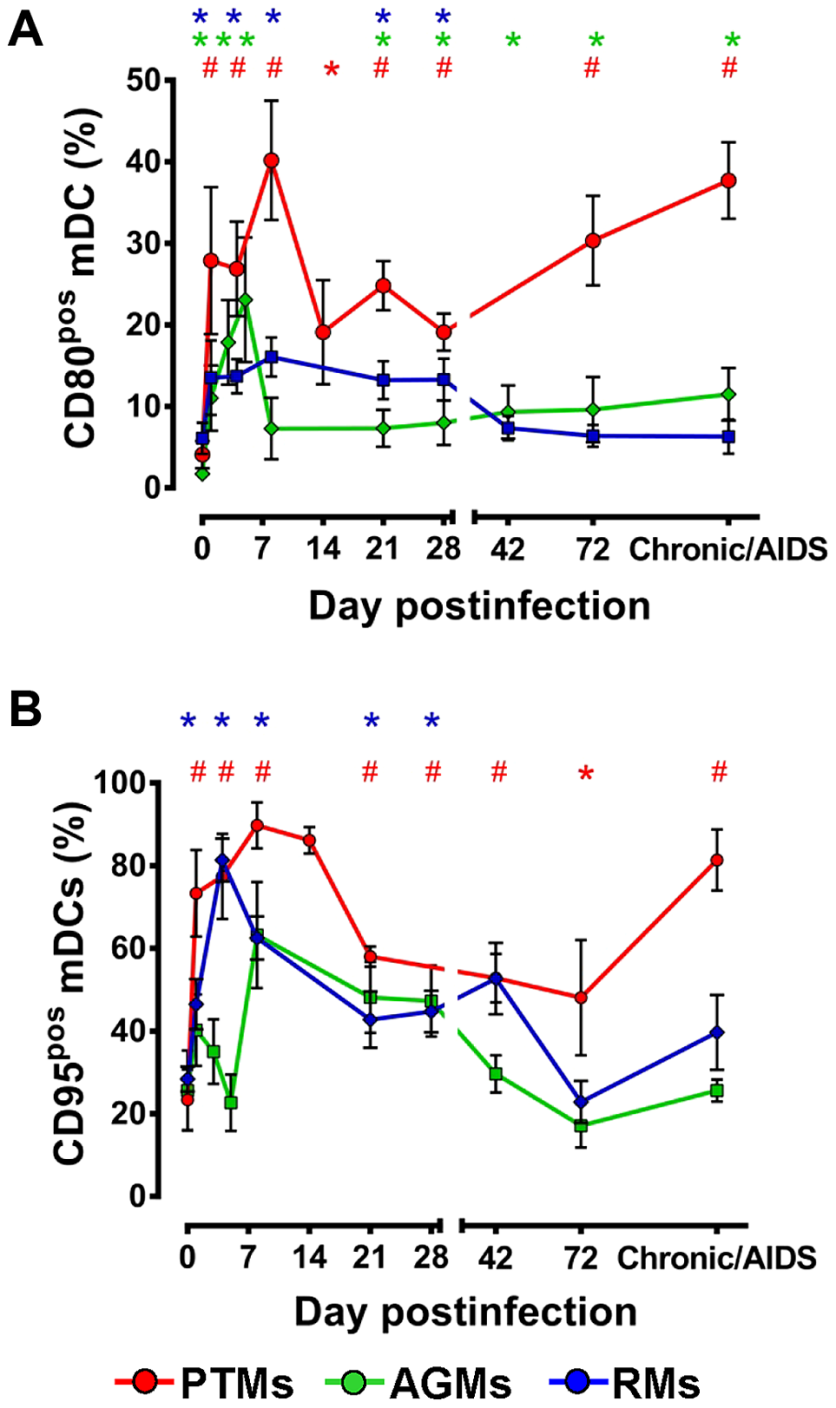
Only pathogenic SIVagmSab infection is characterized by high and persistent mDC activation. (**a**) Dynamics of CD80 expression on circulating mDC in PTMs (red), AGMs (green) and RMs (blue). (**b**) Dynamics of CD95 expression on circulating mDCs in PTMs (red), AGMs (green) and RMs (blue). Shown are mean+SEM. * signifies *p*<0.05; # signifies *p*<0.01 within groups relative to preinfection.

We found that immune activation of mDCs is global and sustained in PTMs, with both CD80 and CD95 being significantly increased throughout follow-up (Figures 8a and b). mDC activation was less significant in AGMs, and limited to increases in CD80 expression during acute and, to a lesser extent, chronic infection, while increases in CD95 expression were not significant in this species (Figures 8a and b). Finally, in SIVagmSab-infected RMs CD80 expression increased on circulating mDCs throughout acute infection and returned to baseline levels with the passage to chronic infection. Similarly, increased CD95 expression on circulating mDCs paralleled that of CD80 in RMs, being limited to the acute infection and returning to baseline during chronic infection (Figure 8b). Interestingly, normalization of mDC activation occurred before complete control of SIVagmSab infection in RMs (Figures 8a and b), in contrast to CD4^+^ T cell activation that normalizes long after the control of viral replication [56].

Collectively, our results identified distinct patterns of mDC immune activation in progressive, nonprogressive and controlled SIV infections that generally parallel the degree of mDC apoptosis. This point to a direct association between the degree of immune activation of innate effectors and their death. This mechanism has also been reported to account for at least a fraction of the CD4^+^ T cell loss during progression to AIDS in pathogenic HIV/SIV infection [37].

### Increased activation and apoptosis of mDCs in progressive infection occurs in response to LPS and TLR7/8 stimulation

**Figure 9:**
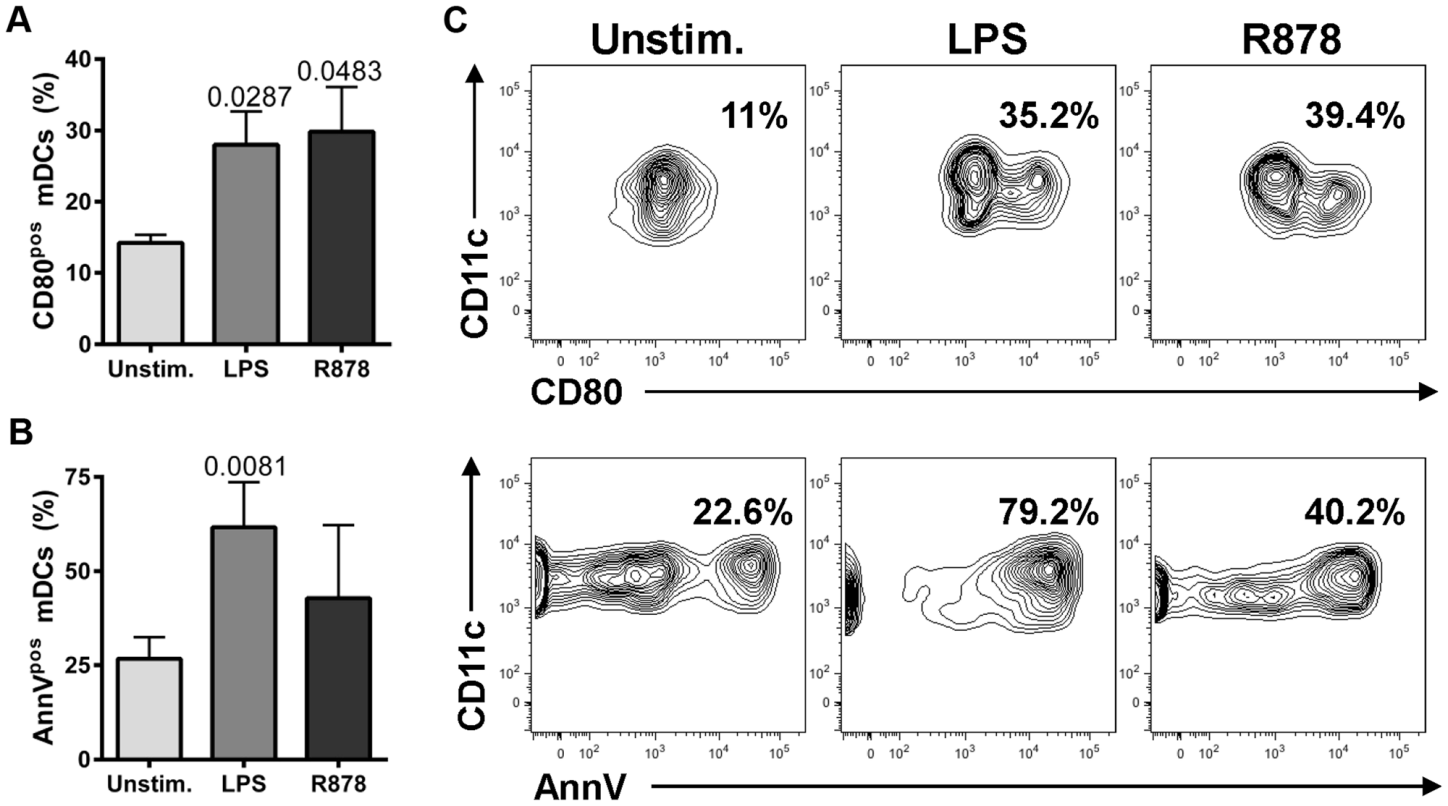
Increased mDC activation and apoptosis occur in PTMs in response to LPS and R848 stimulation. Percentages of peripheral mDCs isolated from uninfected PTMs that express activation markers CD80 (a) or apoptosis marker AnnV (b) in response to LPS and R848 stimulation. Shown are averages+SEM. Representative flow cytometry plots are presented in (c). (Gating was done on live cells and then on Lineage^neg^DR^pos^ CD11c^pos^).

While massive mDC recruitment to intestinal sites occurred in all SIVagmSab disease models, apoptosis associated with increased immune activation of mDCs in both gut and periphery was detected only in the progressive model. We hypothesized that the mDC overstimulation and death unique to SIVagmSab-infected PTMs relies on the severe gut dysfunction, which is characteristic of this progressive infection model. It was reported that translocation of microbial products in the lamina propria and in the general circulation occurs in PTMs [73], where they may enhance mDC stimulation in conjunction with the virus. In contrast, the integrity of the gut is maintained in nonprogressive and controlled infections [73], [74], thus exposing mDCs only to SIV stimulation. We addressed this hypothesis by comparing the ability of LPS (as a surrogate for microbial products) and R848, a TLR7/8 agonist that stimulates mDCs through TLR8, similar to SIV/HIV [75], [76] (as a surrogate for the virus), to induce immune activation and apoptosis in mDCs isolated from PTM blood prior to SIVagmSab infection. This experiment showed that increased immune activation (documented as increased CD80 expression) (Figures 9a and c) and apoptosis [measured as increased expression of Annexin V (AnnV) on the surface of live mDCs] (Figures 9b and c) occurred after stimulation with both LPS and R848. LPS appeared to be a more potent inducer of mDC apoptosis, as shown by the higher levels of AnnV expression on mDCs stimulated with this TLR4 ligand (Figure 9b and c). Altogether, our findings suggest that the combined action of SIV and translocated microbial products may result in excessive activation and apoptosis of circulating and mucosal mDCs in progressive SIV infections.

### Proinflammatory cytokine production by peripheral mDCs in response to TLR7/8 stimulation is decreased in all three animal models after SIV infection

**Figure 10:**
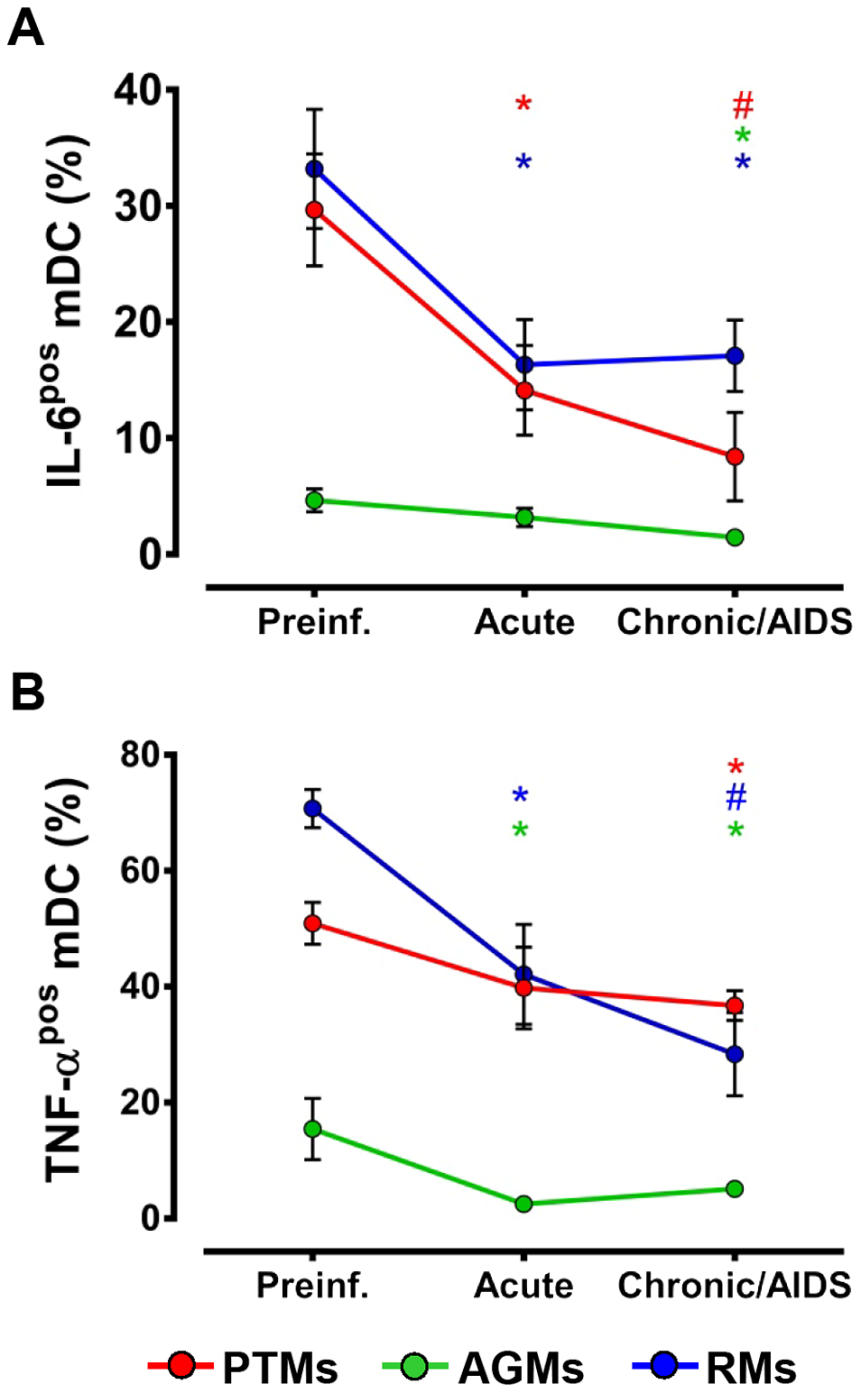
Decreased post-SIVagmSab infection production of proinflammatory cytokines by circulating mDCs stimulated with TLR7/8 ligands. Percentages of peripheral mDCs (collected prior to and after SIVagmSab infection) producing (a) IL-6 and (b) TNF- α in SIVagmSab-infected PTMs (red), AGMs (green) and RMs (blue) after stimulation with TLR7/8 ligands. Shown are mean+SEM. * signifies *p*<0.05; # signifies *p*<0.01 within groups relative to preinfection.

To assess the impact of virus stimulation on mDC function, we compared the cytokine production of mDCs prior to and at critical time points during progressive, nonprogressive and controlled SIV infections. mDCs were isolated and stimulated with R848 [77]. This experiment showed that, within the same species, mDCs from acutely and chronically SIVagmSab-infected monkeys responded to TLR7/8 stimulation by decreasing IL-6 (Figure 10a) and TNF-α production (Figure 10b). The decreased production of proinflammatory cytokines in response to viral stimulation occurred in all three NHP species, independent of the pathogenic outcome of SIVagm infection. Note however, that AGMs exhibited the lowest production of proinflammatory cytokines both before and after SIV infection (Figure 10a and b). These results are in agreement with those previously reported in HIV infection [60], [78] and suggest that SIV infection *per se* does not trigger proinflammatory cytokine production by mDCs.

### Increased proinflammatory cytokine production by circulating mDCs occurs in response to microbial stimulation in progressive SIV infection

**Figure 11:**
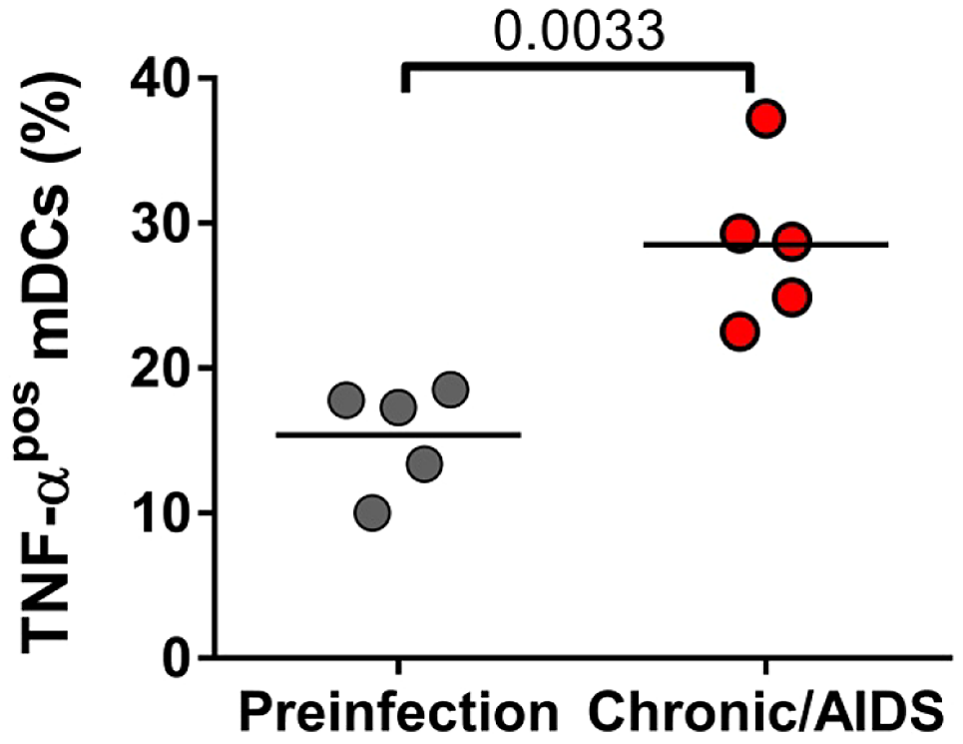
Increased post-SIVagmSab infection production of proinflammatory cytokines by circulating mDCs stimulated with LPS in PTMs. Percentages of peripheral mDCs producing TNF-α in PTMs prior to and during chronic SIVagmSab infection.

Since microbial translocation (MT) significantly increases only in pathogenic SIVagmSab infection of PTMs and not in nonprogressive SIVagmSab infection of AGMs and RMs [42], [56], we reasoned that mDCs might be stimulated to produce proinflammatory cytokines by the microbial products translocated from the gut during pathogenic SIV infections. We assessed TNF-α production in response to LPS stimulation of peripheral mDCs isolated from PTMs prior to SIV infection and during chronic infection. Our measurements revealed that TNF production is indeed increased in LPS-stimulated mDCs isolated from chronically SIV-infected PTMs (Figure 11), indicating that mDCs may contribute to the increased immune activation and inflammation observed during progressive SIV/HIV infection.

### Spontaneous proinflammatory cytokine production by intestinal mDCs is increased only during chronic progressive SIVagmSab infection of PTMs

**Figure 12:**
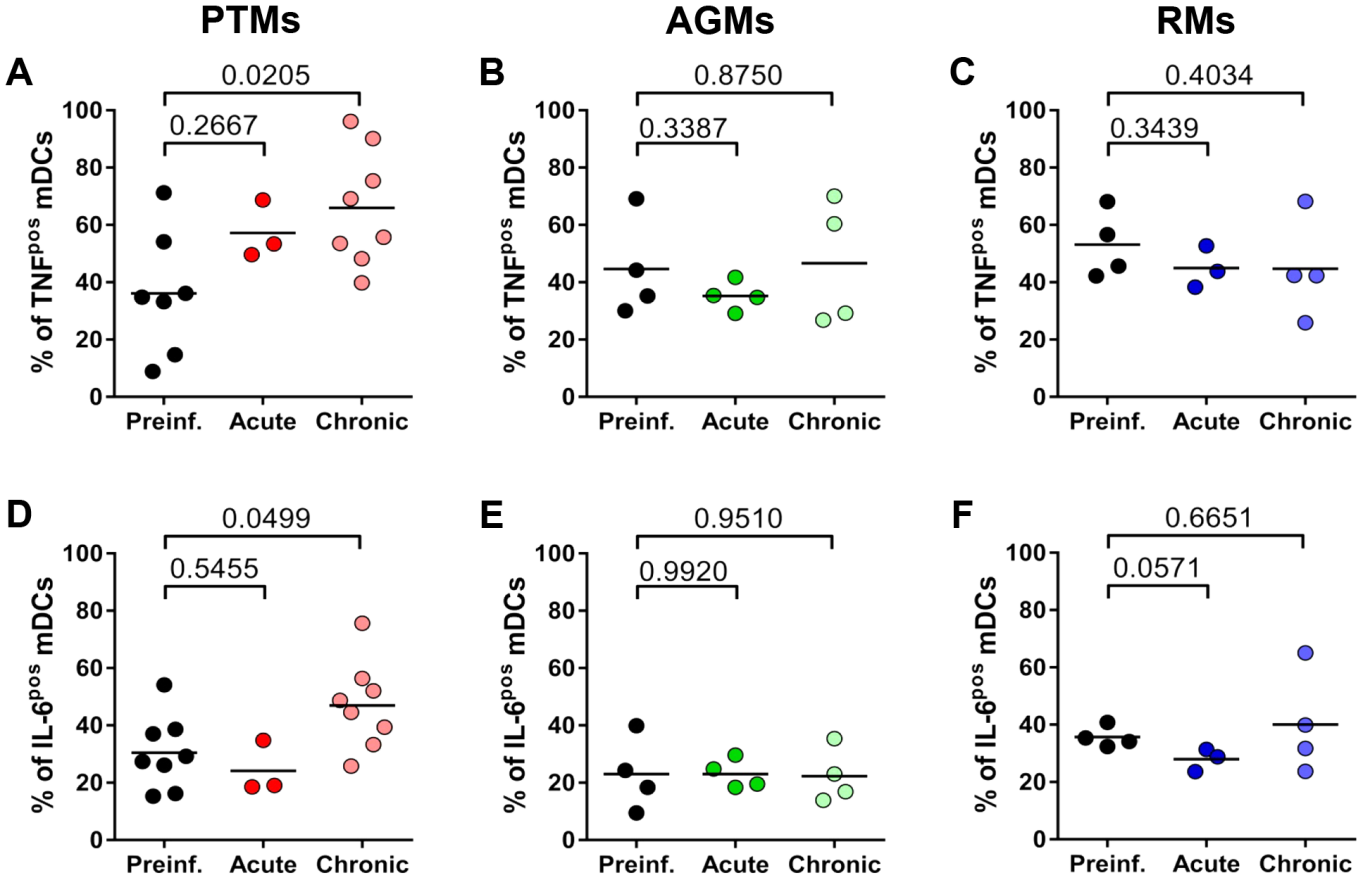
Increased post-SIVagmSab infection production of proinflammatory cytokines by intestinal unstimulated mDCs occurs only in progressive infection. Percentages of intestinal unstimulated mDCs producing TNF-α in (a) PTMs, (b) AGMs and (c) RMs prior to and during acute and chronic SIVagmSab infection. Percentages of intestinal unstimulated mDCs producing IL-6 in (d) PTMs, (e) AGMs and (f) RMs prior to and during acute and chronic SIVagmSab infection. Gating was done on live cells and then on Lineage^neg^ HLA-DR^pos^ CD11c^pos^.

To determine if mDCs contribute to the gut dysfunction described in progressive HIV/SIV infections, we compared the spontaneous production of proinflammatory cytokines (TNF-α and IL-6) by unstimulated mDC isolated from the gut in normal *vs.* acutely and chronically SIVagmSab-infected PTMs. We also compared and contrasted these results with those obtained from assessment of the function of unstimulated intestinal mDCs isolated from normal, acutely and chronically SIVagmSab-infected AGMs and RMs which lack intestinal dysfunction [56], [73]. No significant difference in cytokine production was observed in any of the three models between acutely infected and uninfected animals (Figure 12).

TNF-α and IL-6 production by intestinal mDCs isolated from chronically SIVagmSab-infected PTMs was significantly greater than that of intestinal mDCs from uninfected PTMs (Figure 12a and d). No modifications in mDC TNF-α and IL-6 production could be detected in chronically infected AGMs (Figure 12b and e) and RMs (Figure 12c and f).

Our results suggest that intestinal mDCs play a significant role in the etiology of the gut dysfunction characteristic of progressive infection. Furthermore, increased mDC production of TNF-α in the intestine is not beneficial and is associated with a poor clinical prognosis, in agreement with previous reports [79]. Finally, these data support our hypothesis that mDC overstimulation and increased proinflammatory cytokine production occurs only in the animal model in which microbial products are translocated into the lamina propria.

## Discussion

In this study, we assessed the role of mDCs in the pathogenesis of HIV/SIV infection and AIDS. The rationale for focusing on this cell subset is that mDCs are major regulators of immune activation and inflammation [2], [3], two major determinants of HIV disease progression [80]. A limitation of previous mDC studies is that they have exclusively focused on pathogenic HIV/SIV infections in which a major depletion of circulating mDCs occurs, thus precluding the assignment of a major role to this subset in driving or containing disease progression. Conversely, comparing and contrasting the fate of mDCs in pathogenic, nonpathogenic and controlled SIV infections allowed us to dissect the role of mDCs in SIV pathogenesis.

For these comparative studies, we used three different NHP models developed in our lab, in which the same viral strain, SIVagmSab, induces different pathogenic outcomes: persistent progressive (pathogenic) infection in PTMs [81], [82], persistent nonprogressive (nonpathogenic) infection in AGMs [61], [83] and completely controlled infection in RMs [56]. The use of the same SIVagmSab strain to produce these different pathogenic outcomes eliminated the interference of viral factors on pathogenesis while permitting the targeting of host responses with significant impact on the outcome of infection. Thus, our biological model allows us to complement the existing information regarding the fate of mDCs during pathogenic infection with data from nonpathogenic and elite-controlled infections and to address two fundamental questions: (i) what is the mechanism of mDC depletion in HIV/SIV infection? and (ii) what is the role of mDCs in the pathogenesis of SIV infection? We performed a stepwise approach to address these key issues:

### What is the overall significance of mDC depletion for the outcome of SIV infection?

We report that, in agreement with previous reports [7], a significant drop in peripheral mDC counts occurs during the pathogenic SIV infection of PTMs. However, our comparative analysis also identified a similar depletion of circulating mDCs in the nonpathogenic infection of AGMs. As such, our study suggests that the magnitude of the acute depletion of circulating mDCs is not predictive of SIV disease progression. Similarly, while it was previously reported that mDC counts at the setpoint are predictive of the outcome of infection [84], our results could not establish such a correlation.

In our study, mDC levels during chronic infection appear to be associated with the control of disease progression. Thus, in RMs, mDC levels increased over baseline levels coincident with the control of viral replication. Altogether, these features point to mDC restoration as one of the mechanisms through which control of SIV infection is achieved.

### Is mDC depletion from circulation due to direct killing by SIV?

Our results suggest that mDC depletion from circulation is not due to direct virus killing: (i) The three species exhibited similar high levels of viral replication during acute infection (Figure 1), yet mDCs were depleted only in PTMs and AGMs, but not in RMs. (ii) The kinetics of acute mDC depletion from circulation significantly diverged between PTMs and AGMs, in spite of similar dynamics of viral replication. (iii) mDC depletion occured prior to detectable viremia in PTMs. (iv) SIVagmSab does not have a *vpx* gene and therefore cannot infect dendritic cells because of the SAMHD1 restriction [64], as documented in our study that failed to identify viral DNA in sorted mDCs from SIVagmSab-infected PTMs, AGMs and RMs. By documenting mDC depletion in a biological system that uses a virus that does not infect mDCs, our study demonstrates that factors other than direct virus killing are responsible for mDC depletion during SIV/HIV infection.

### Is depletion of circulating mDCs due to insufficient mobilization from the bone marrow?

We assessed mDC mobilization from the bone marrow by measuring Ki-67 expression. For the same purpose, other groups have used thymidine analogues such as Bromodeoxyuridine (BrdU), which becomes incorporated into dividing mDCs. BrdU assesses mDC mobilization from the bone marrow and tracks the cells to tissues. However, BrdU induces mutations, limiting its use in long-term studies in large animal models [85]. Furthermore, studies reported that BrdU incorporation and Ki-67 expression in DCs have a similar value [86]. Therefore, we reasoned that measuring the expression of cell proliferation markers by mDCs is appropriate to assess trafficking in long-term studies and postulated that Ki-67 expression in tissue mDCs is due to rapid recruitment of cells that were recently mobilized into circulation from the bone marrow.

We report that mDC mobilization from the bone marrow occurs in pathogenic, nonpathogenic and controlled SIV infections. Increased mobilization was observed throughout the follow-up and did not appear disrupted during the terminal stages of infection in the animals that progressed to AIDS. Furthermore, rapid progression to AIDS was not associated with a defect in mDC mobilization from the bone marrow. As such, our study failed to document any evidence that insufficient mobilization of mDCs from the bone marrow is a factor driving the drop of circulating mDCs.

### Is mDC depletion from circulation due to migration to other sites?

We took a series of steps to carefully assess mDC migration to the LNs and the gut. By immunophenotyping the mDCs from the LNs and intestine, we showed that increases of mDCs in the LNs and intestine occur in persistent nonprogressive SIVagm infection in AGMs and to a lesser extent in controlled SIVagm infection in RMs, suggesting mDC recruitment to these sites. Conversely, transient loss of mDCs from LNs and intestinal mucosal sites only occurred in SIVagm-infected PTMs. To explain this discrepancy between progressive and nonprogressive infections, we further assessed Ki-67 expression by mDCs at the LNs and mucosal sites, as a sign of their recent mobilization, and we showed that Ki-67 expression increased in all models throughout infection, suggesting massive mDC influx at these sites. Next, we assessed mDC surface expression of the chemokine receptors involved in cell mobilization to LNs [66]–[68] and mucosal [69] tissues to further document mDC mobilization to these sites in the three types of infections. Increased levels of CCR5 and CCR7 documented a massive influx of mDCs to both LNs and mucosal tissues in the pathogenic infection of PTMs. Conversely, in the nonpathogenic infection of AGMs, a low expression of CCR5 by the mDCs recapitulates previous reports in this species documenting a similar low CCR5 expression by CD4^+^ T cells [44], [45] and suggests a reduced mDC migration to the intestine in this animal model. Meanwhile, transient increases of both CCR5 and CCR7 pointed to a brief mDC mobilization to both LNs and intestine in SIVagm-infected RMs and showed that mDC trafficking to these tissues returns to baseline when viral replication is controlled. We also assessed the expression of the intestine homing marker α4β7 integrin on mDCs and documented their migration to the gut in PTMs and AGMs. Finally, the flow cytometry results were confirmed by IHC. Altogether, our data demonstrate here for the first time that mDC mobilization to the LNs and mucosal tissues occurs in progressive, nonprogressive and controlled SIVagmSab infections and is a major factor responsible for their depletion from circulation.

### What is the mechanism of mucosal mDC loss in pathogenic SIV infection?

mDCs were either depleted or not significantly increased from the intestine during pathogenic infection, in spite of continuous recruitment to this site. As such, we assessed the expression of apoptosis markers by mDCs in progressive SIV infection of PTMs and demonstrated that mDC depletion is indeed due to increased programmed cell death. By comparing the results of *in vitro* stimulation of mDCs isolated from uninfected PTMs with R848 (to mimic virus stimulation) and LPS (a surrogate for microbial products that are abundantly present in the lamina propria and general circulation in SIV-infected PTMs), we found that mDC hyperactivation and death is due to microbial products rather than the virus. These data corroborate our findings that a lower degree of mDC activation and no significant increase in apoptosis are associated with lack of MT, rather than low viremia, in the nonprogressive infections.

While it is difficult to extrapolate *in vivo* the *in vitro* data and conclusively establish which is *the* major cause of mDC hyperactivation and death during SIV infection, our data supports a view in which a synergistic SIV and microbial mDC hyperstimulation are responsible for the depletion of this immune cell subset in pathogenic HIV/SIV infections. Conversely, in nonprogressive and controlled infections, mDC apoptosis may be kept at bay because, in these models, there is no MT associated with SIVagmSab infection and mDC stimulation occurs predominantly through direct virus action.

### What is the role of mDCs in the pathogenesis of HIV/SIV infection and progression to AIDS?

This is a key question addressed by our study. Our complex approach allowed us to establish four lines of evidence in support of a positive role for mDCs in SIV infection: (i) We established a direct correlation between mDC counts and prognosis of SIV infection: animals with the lowest mDC levels prior to infection were rapid progressors; mDC depletion from blood and intestine was associated with disease progression; animals that completely recovered mDCs during chronic infection experienced delayed or no disease progression. (ii) While mDC mobilization from the bone marrow occurred in all the NHPs included in our study, increased mobilization was associated with a lack of disease progression during the follow up (Figure 4), suggesting possible mDC involvement in virus control. (iii) We documented mDC mobilization to LNs and intestine in all three species in response to SIV infection and showed that increased levels of mDCs in the intestine and LNs are associated with lack of disease progression/control of infection, while mDC loss at these sites is associated with progression to AIDS. (iv) The comparison between progressive, nonprogressive and controlled infections revealed that mDCs are not inducing immune activation and inflammation in the biological systems in which SIV replication is not associated with MT. Thus, mDC accumulation in the intestine in nonprogressive and controlled SIVagmSab infection of AGMs and RMs, respectively, is not associated with increases in immune activation and inflammation in these species. This is supported by our findings that peripheral mDC production of proinflammatory cytokines in response to TLR7/8 ligands after SIV infection is decreased in these species. Adequate preservation of mDCs may impact immune activation and inflammation in SIVagmSab-infected AGMs and RMs through additional pathways. It was recently reported that subsets of mDCs may play a significant role in inducing differentiation of Tregs, which may in turn control immune activation and promote tolerance to SIV infection [87]. Altogether, these features strongly support a role of mDCs in reducing the levels of immune activation and inflammation, maintaining gut integrity and possibly inducing tolerance to SIV in nonprogressive infections.

Due to their depletion from both circulation and the intestine, it is virtually impossible to precisely define the role of mDCs in progressive infection. While our studies showed that mDCs isolated from PTMs show a similar hyporesponsiveness postinfection to TLR7/8 stimulation compared to nonprogressive infections, mDC activation was significantly higher in PTMs. Our results show that mDC hyperactivation and production of proinflammatory cytokines may be enhanced in progressive SIV infection by microbial products translocated to the circulation as a result of the severe SIV-associated gut dysfunction in these species. Finally, we documented increased TNF-α and IL-6 production by intestinal mDCs isolated from PTMs (Figure 12), suggesting an association between increased inflammatory cytokine production and poor clinical prognosis. Altogether, our results show that during progressive SIV/HIV infections, the beneficial role of mDCs may be obscured by either their excessive loss and/or by hyperfunction reflected in an overproduction of proinflammatory cytokines by residual mDCs in response to microbial products or opportunistic pathogens concurrent with HIV/SIV infection.

In conclusion, we report that mDCs have different kinetics, immune activation/apoptotic patterns and functions in pathogenic, nonpathogenic and controlled SIV infection. Our results provide a mechanistic basis of the role of mDCs in the pathogenesis of AIDS. These results are informative for designing both therapeutic interventions and vaccinations aimed at controlling HIV infection.

## Methods

### Ethical statement

All animals were housed and maintained at the University of Pittsburgh according to the standards of the Association for Assessment and Accreditation of Laboratory Animal Care (AAALAC), and experiments were approved by the University of Pittsburgh Institutional Animal Care and Use Committee (IACUC). These studies were covered by two IACUC protocols: 0907039/12080831 Animal Model for SIV Infection Control (Approved in 2009 and renewed in 2012); 0911844/12121250 Pathogenesis of SIV in African green monkeys (Approved in 2009 and renewed in 2012). The animals were fed and housed according to regulations set forth by the *Guide for the Care and Use of Laboratory Animals* and the Animal Welfare Act.

### Animals and infections

Four AGMs, five RMs and five PTMs were included in the study. All animals were infected with plasma equivalent to 300 tissue culture infectious doses (TCID50) of SIVagm.sab92018 [61] and followed for up to one year or until progression to AIDS. Animals were clinically monitored throughout the follow-up. Plasma VLs were quantified by real time-PCR as previously described [61].

### Sampling and sample processing

Blood was collected from all animals at the following time points: prior to the infection (0 dpi), during acute SIVagm infection to closely monitor the innate responses (1, 3, 4, 5, 8, 14, 21 and 28 dpi), around the viral setpoint (35 and 42 dpi), and during chronic infection (60, 72, and 120 dpi).

LN biopsies were performed prior to infection, at the peak of viral load (during acute infection), and at necropsy (during chronic infection). Intestinal resections (5–10 cm) were surgically performed prior to infection, during acute infection and during chronic infection, as previously described [42], [62]. Additional intestinal samples were collected at the necropsy.

Within one hour after blood collection, plasma was harvested and peripheral blood mononuclear cells (PBMCs) were separated from the blood using Ficoll density gradient centrifugation. Lymphocytes from the intestine and LNs were isolated and stained for flow-cytometry, as previously described [42], [61], [62]. Lymphocytes were isolated from the axillary or inguinal LNs by gently mincing and pressing tissues through nylon mesh screens. Intestinal resections were processed as described previously to obtain an enriched mononuclear cell suspension [42], [61], [62], [88]. Briefly, intestinal resections were minced mechanically, washed with EDTA and subjected to collagenase digestion followed by Percoll density gradient centrifugation.

PBMCs, LNs and intestinal cells from PTMs, AGMs and RMs infected through the same route and with the same dose of SIVagm.sab as in previous protocols were also included to match infection time points when samples from the present study were not available, or to increase the number of animals and improve statistical significance.

### Flow cytometry

Whole peripheral blood, LN cell suspensions and intestinal mononuclear cell suspensions were stained with fluorescently-labeled antibodies to Lineage markers CD3 (clone SP34-2; all antibodies from BD Bioscience, San Jose, CA, USA unless otherwise noted), CD14 (M5E2), CD163 (GH1/6, only in LN and intestine) and CD20 (2H7)]; HLA-DR (G46-6), CD11c (S-HCL-3), CD103 (2G5) CD45 (D058-1283), CD80 (L307.4), CD86 (FUN-1), CD95 (DX2), CCR5 (3A9), CCR7 (3D12, eBioscience, San Diego, CA, USA) and α4β7 (FIB504). An amine-reactive fixable dead-cell dye (Invitrogen, Grand Island, NY, USA) was used to distinguish live from dead cells. For intracellular staining, cells were stained to identify live mDCs as described above, then fixed, permeabilized and stained for activated Caspase-3 (Cas-3, C92-605) and Ki-67 (B56). Flow cytometry acquisitions were performed on an LSR II flow cytometer and analyzed with FlowJo software (Treestar, Ashland, OR, USA).

mDCs were defined as CD45^+^ (only in peripheral blood) Lineage negative (Lineage^neg^) HLA-DR^+^ cells expressing CD11c (Figure 2). In the LNs, a broad Lineage^neg^ HLA-DR^+/++^ gate was used to include all mDCs, as described previously (Figure 2). mDC populations positive for CD80, CD86, CD95, CCR5, CCR7, α4β7 and Ki-67 were identified by staining with an isotype control antibody. Cas-3^+^ mDCs were identified as a distinctly separate population.

A two-step TruCount technique was used to enumerate mDCs in blood, as previously reported [89]. The number of blood CD45^+^ cells was quantified using a precise volume of blood stained with antibodies in TruCount tubes (BD Biosciences) that contained a defined number of fluorescent beads to provide internal calibration. The number of blood mDCs was then calculated based on the ratio of mDCs to CD45 cells in whole blood at the same time point.

### mDC, pDC, and macrophage sorting, nucleic acid extraction, and viral copy number quantification

Cryopreserved single-cell preparations of superficial lymph nodes collected at time of necropsy from various protocols were thawed and stained with CD3/C20-PE, CD14-FITC, HLA-DR-APC-Cy7, CD11c-APC, CD123-PE-Cy7, Live/Dead-Yellow Dye L-34959 from Molecular Probes (Eugene, OR) for 30 minutes at 4°C; then washed once with and resuspended in PBS supplemented with 0.5% BSA and 2 mM EDTA. mDCs (Lineage^neg^ CD14^neg^ HLA-DR^+^ CD123^neg^ CD11c^+^) along with Lineage^+^ cells, were sorted on a 3-laser FACSAria instrument using FACSDiva 6 software (BD Biosciences) and collected in polystyrene tubes containing RPMI+20% heat-inactivated FBS. The sorted cells were pelleted and frozen, and DNA was extracted with the DNeasy Blood and Tissue Kit (Qiagen, Germantown, MD). Viral copy number was determined by qPCR of extracted DNA, as previously described [56].

### Immunohistochemical (IHC) assessment of mDCs using CD11c mAb

IHC was performed on formalin-fixed, paraffin-embedded tissue samples. Four µm thick sections were deparaffinized, rehydrated, and rinsed. For antigen retrieval, the sections were microwaved in Vector Unmasking Solution and treated with 3% hydrogen peroxide. Sections were incubated with CD11c monoclonal primary antibody (Novacastra, USA). Secondary antibodies were from Vector Vectastain ABC Elite Kit. For visualization, sections were treated with DAB (Dako) and counterstained with hematoxylin.

### In vitro mDC stimulation

mDC activation and apoptosis was measured after stimulation of total PBMCs for 24 hours with 10 µM of the TLR7/8 ligand R848, (Invivogen, San Diego, CA, USA) or with 100 ng/ml *Escherichia coli* lipopolysaccharide (LPS), (Invivogen, San Diego, CA, USA). Cells were harvested and stained for dendritic cell markers, CD80 and AnnV. An amine-reactive fixable dead-cell dye (Invitrogen, Grand Island, NY, USA) was used to discriminate live from dead cells. Cells cultured in the absence of R848 and LPS were used as background control.

### Intracellular cytokine assays

Intracellular cytokine production by isolated mononuclear cells from blood and intestine was measured as described previously, with slight variation [7]. Briefly, cells were cultured for seven hours with 10 µM of the TLR7/8 ligand R848 (Invivogen, San Diego, CA, USA) or with *Escherichia coli* lipopolysaccharide (LPS; 100 ng/ml; Invivogen, San Diego, CA, USA) with and without the addition of 10 µg/mL brefeldin A (Sigma, St. Louis, MO, USA) after two hours. Cells were stained with surface-labeling antibodies as above then fixed and permeabilized prior to incubation with antibodies to TNF-α (MAb11), IL-6 (MQ2-6A3) and IL-12 (C8.6, eBioscience) and analyzed by flow cytometry. Cells (mDCs) cultured with R848 or LPS stimulation but without the addition of brefeldin A were used as background control.

### Statistical analysis

In each species, postinfection time point values for each parameter were compared with pre-infection values using the Mann-Whitney U test. GraphPad Prism 5 (GraphPad Software) was used for statistical analysis. Correlations were determined using the non-parametric Spearman rank test. Differences in temporal dynamics were analyzed using mixed-effects models, with macaque as the grouping factor to account for the repeated measurements made in each animal. For these analysis we used the nlme package [90] of R (http://cran.r-project.org/). All P<0.05 values were considered to be significant.

## Supporting Information

Figure S1 **SIVagmSab rapid disease progression is predicted by lower counts of circulating mDC prior to SIV infection.** Comparison of the baseline mDC levels between normal progressor (black circles) and rapid progressor (red squares) pigtailed macaques.
(TIFF)

Figure S2 **SIVagmSab disease progression in PTMs is associated with significant loss of circulating mDCs.** Comparison of mDC levels prior to infection and during the late chronic SIVagmSab infection. Included are both the PTMs used in this study and historic samples from SIVagmSab-infected PTMs.
(TIFF)

Figure S3 **Rapid progression of SIVagmSab infection in PTMs is not characterized by a defect in mDC mobilization from the bone marrow.** Dynamics of Ki-67-expressing mDCs are shown in blood in SIVagm-infected PTMs, rapid progressors (red) and normal progressors (black).
(TIF)

Figure S4 **CD103^pos^ mDC are preferentially depleted in the intestine of SIVagmSab-infected PTMs.** (a) The classic CD11c^pos^ mDC population is not significantly depleted in the gut in chronically SIVagmSab-infected PTMs. (b) Mucosal CD103^pos^ mDCs are lost in chronically SIV-infected PTMs.
(TIFF)

Figure S5 **Immunohistochemical assessment of CD11c expression in the superficial (upper panels) and mesenteric (lower panels) LNs collected from SIVagmSab-infected PTMs.** LNs were collected prior to infection (a and d), during acute infection (b and e) and during the chronic stage of infection (c and f). CD11c expression is decreased during acute infection (b) in the superficial LNs compared to preinfection (a) and chronic infection (c). In the mesenteric LNs, CD11c expression is increased during chronic SIVagmSab infection (f) compared to preinfection (d) and acute infection (e). Magnification: 10×.
(TIFF)

Figure S6 **Immunohistochemical assessment of CD11c expression in the jejunum samples collected from SIVagmSab-infected PTMs.** CD11c expression is decreased during acute infection (b) compared to preinfection (a) and chronic infection (c). Magnification: 10×.
(TIFF)

Figure S7 **Immunohistochemical assessment of CD11c expression in tissues collected from SIVagmSab-infected AGMs.** No difference in the expression of CD11c in the LNs collected prior to infection (a), as well as during acute (b) and chronic (c) SIVagmSab infection. CD11c expression in the Peyer's patches (f) and in the lamina propria (i) increased during the chronic stage of infection compared to preinfection levels (d and g) and those observed during the acute infection (e and h). Magnification: 10×.
(TIFF)

Figure S8 **Immunohistochemical assessment of CD11c expression in tissues collected from SIVagmSab-infected RMs.** No difference in the expression of CD11c in the LNs collected prior to infection (a), as well as during acute (b) and chronic (c) SIVagmSab infection. CD11c expression increased in the lamina propria during acute infection (e and h) compared to preinfection levels (d and g) and those observed during the chronic infection (f and i). The CD11c^+^ cells are mainly present in the lymphoid follicles (d–f). Magnification: 10×.
(TIFF)

Figure S9 **mDC mobilization to the intestine occurs in pathogenic (PTMs) SIVagm infection.** Assessment of α4β7 expression on circulating mDCs in SIVagm-infected PTMs (red), AGMs (green) and RMs (blue). # signifies *p*<0.01 changes from the baseline levels within the same animal group (Anova).
(TIF)
