# Supplementary figures and images for: Kinetics of Myeloid Dendritic Cell Trafficking and Activation: Impact on Progressive, Nonprogressive and Controlled SIV Infections
Source: PLoS Pathog. 2013 Oct 3;9(10):e1003600. doi: 10.1371/journal.ppat.1003600 (PMC3789723; doi:10.1371/journal.ppat.1003600)

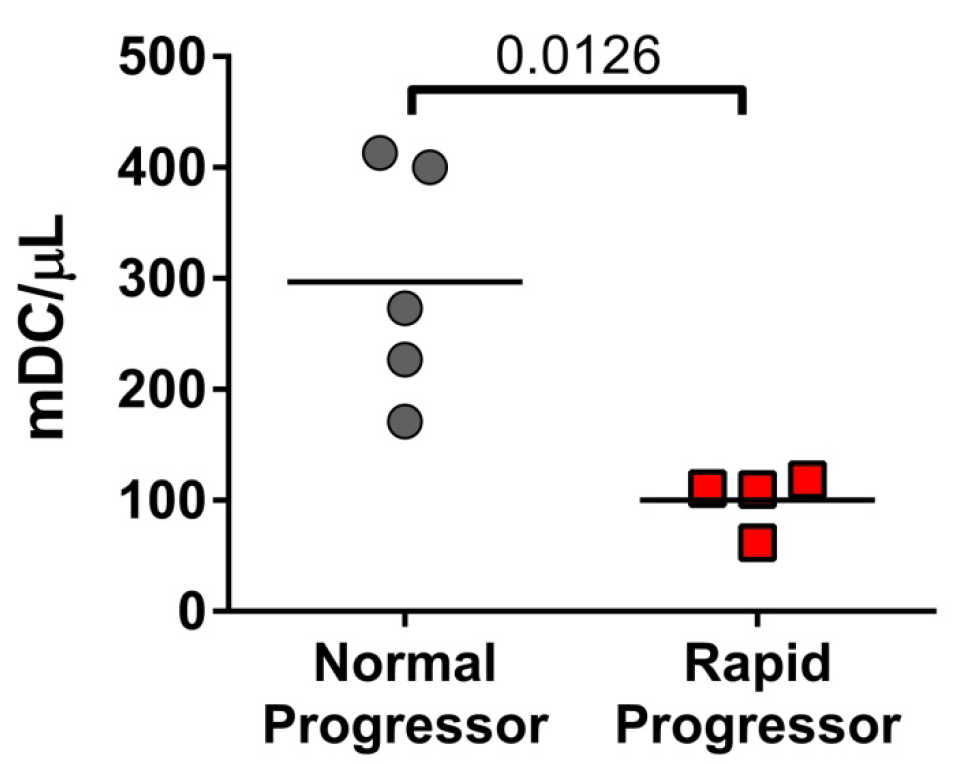

Supplement: Figure S1 — SIVagmSab rapid disease progression is predicted by lower counts of circulating mDC prior to SIV infection. Comparison of the baseline mDC levels between normal progressor (black circles) and rapid progressor (red squares) pigtailed macaques. (TIFF) [file ppat.1003600.s001.tiff]

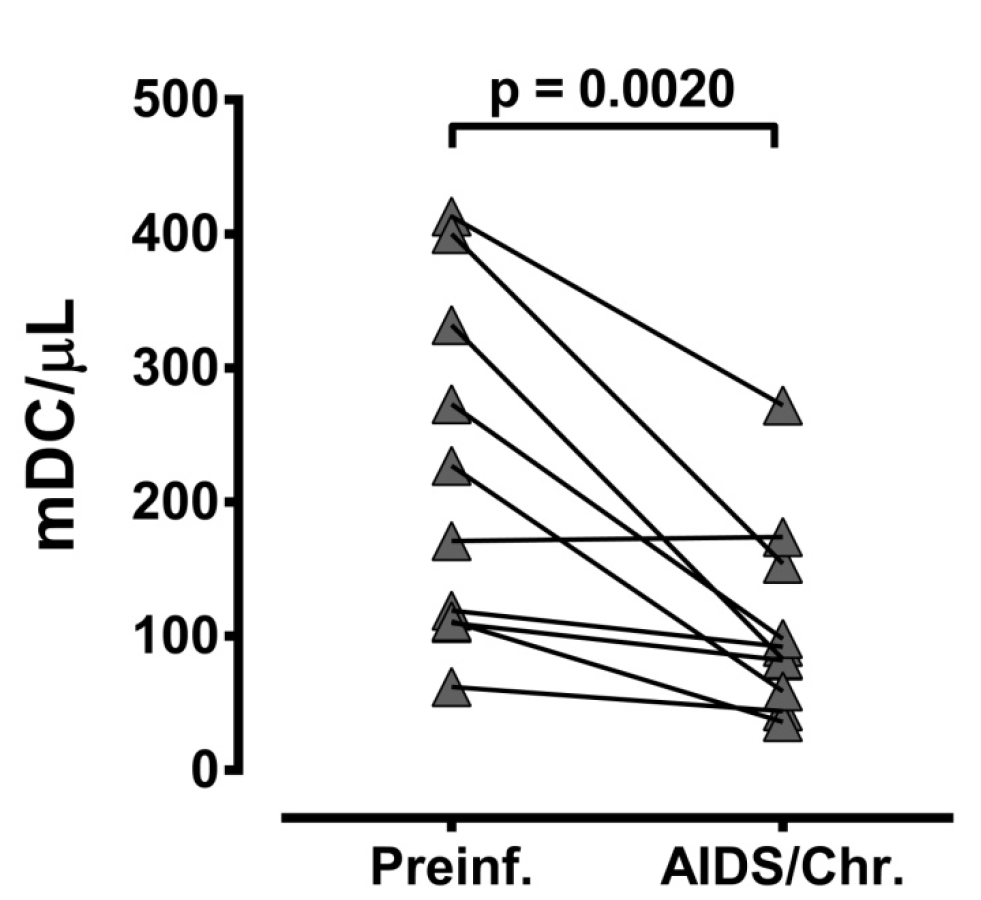

Supplement: Figure S2 — SIVagmSab disease progression in PTMs is associated with significant loss of circulating mDCs. Comparison of mDC levels prior to infection and during the late chronic SIVagmSab infection. Included are both the PTMs used in this study and historic samples from SIVagmSab-infected PTMs. (TIFF) [file ppat.1003600.s002.tiff]

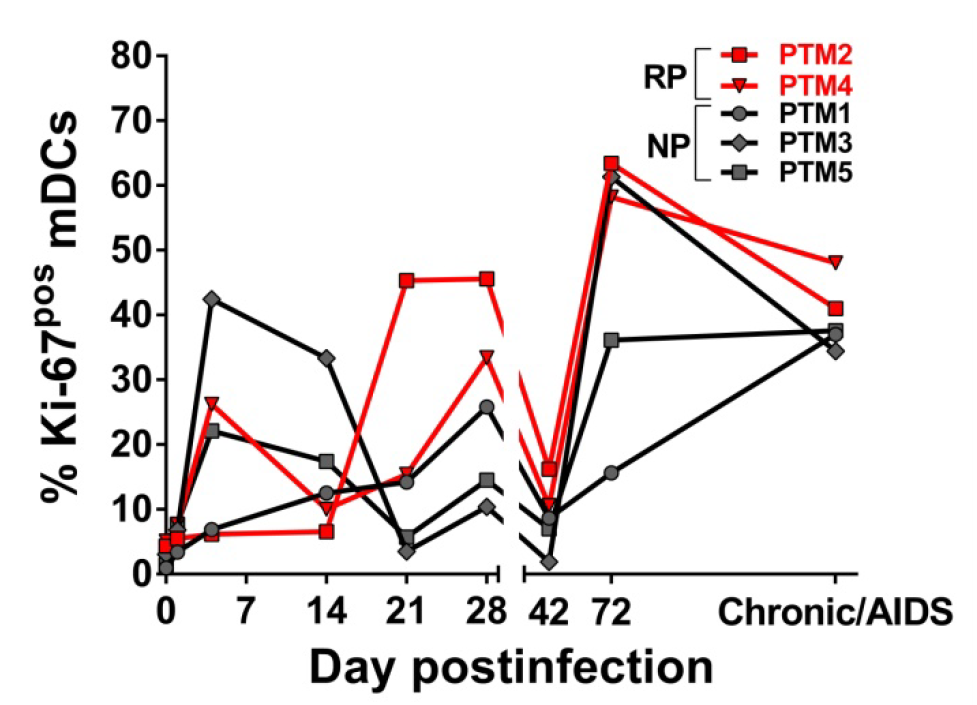

Supplement: Figure S3 — Rapid progression of SIVagmSab infection in PTMs is not characterized by a defect in mDC mobilization from the bone marrow. Dynamics of Ki-67-expressing mDCs are shown in blood in SIVagm-infected PTMs, rapid progressors (red) and normal progressors (black). (TIF) [file ppat.1003600.s003.tif]

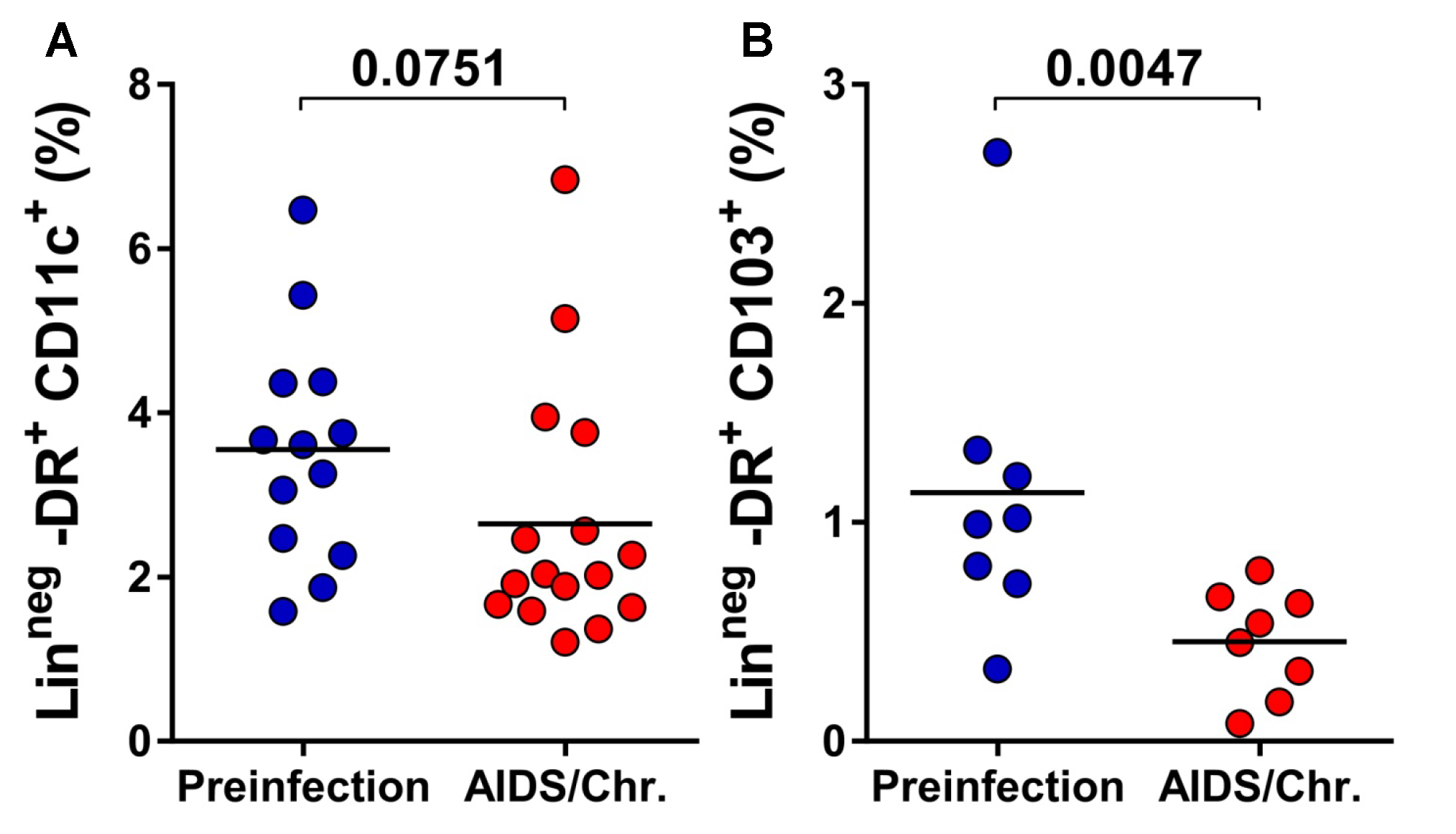

Supplement: Figure S4 — CD103pos mDC are preferentially depleted in the intestine of SIVagmSab-infected PTMs. (a) The classic CD11cpos mDC population is not significantly depleted in the gut in chronically SIVagmSab-infected PTMs. (b) Mucosal CD103pos mDCs are lost in chronically SIV-infected PTMs. (TIFF) [file ppat.1003600.s004.tiff]

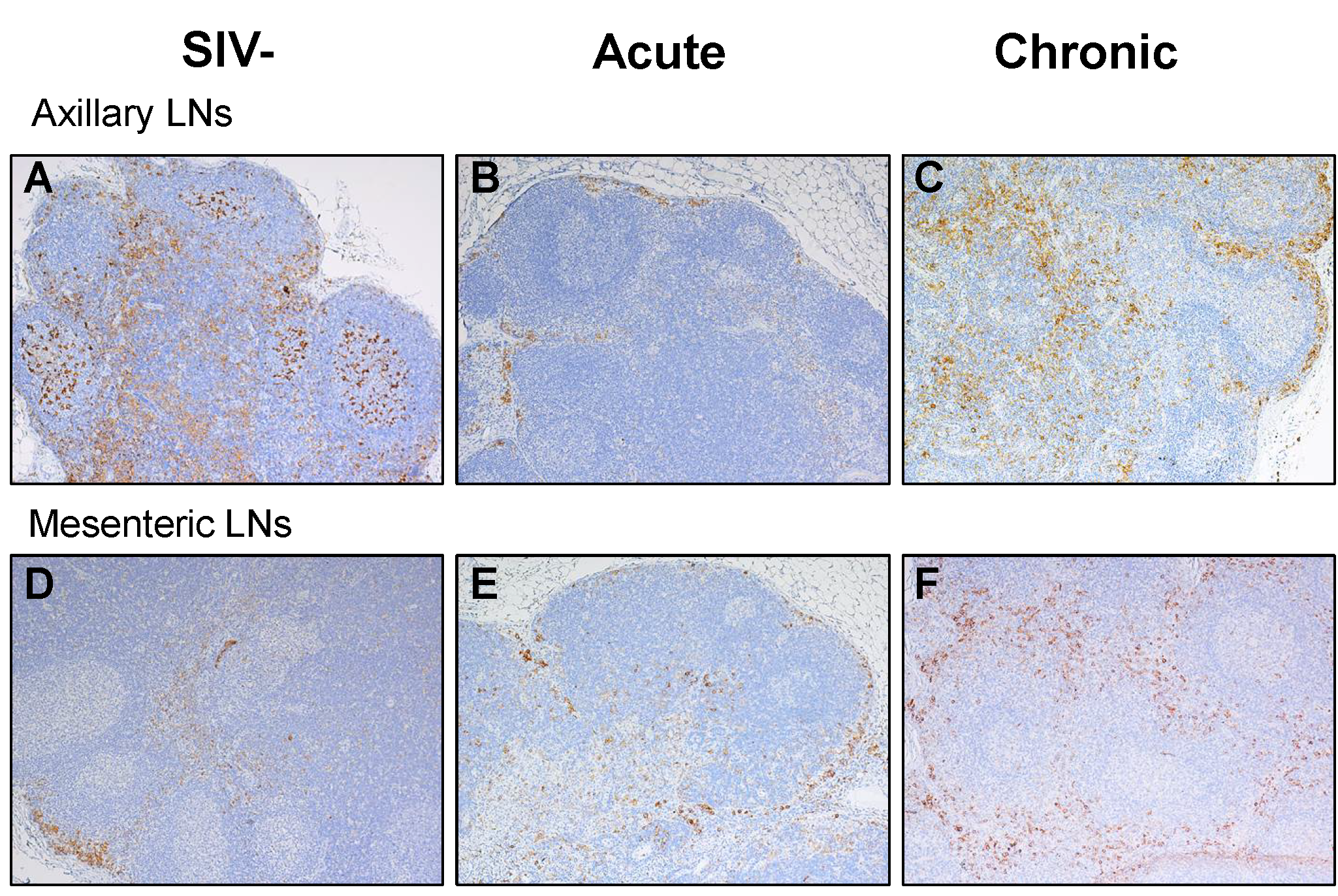

Supplement: Figure S5 — Immunohistochemical assessment of CD11c expression in the superficial (upper panels) and mesenteric (lower panels) LNs collected from SIVagmSab-infected PTMs. LNs were collected prior to infection (a and d), during acute infection (b and e) and during the chronic stage of infection (c and f). CD11c expression is decreased during acute infection (b) in the superficial LNs compared to preinfection (a) and chronic infection (c). In the mesenteric LNs, CD11c expression is increased during chronic SIVagmSab infection (f) compared to preinfection (d) and acute infection (e). Magnification: 10×. (TIFF) [file ppat.1003600.s005.tiff]

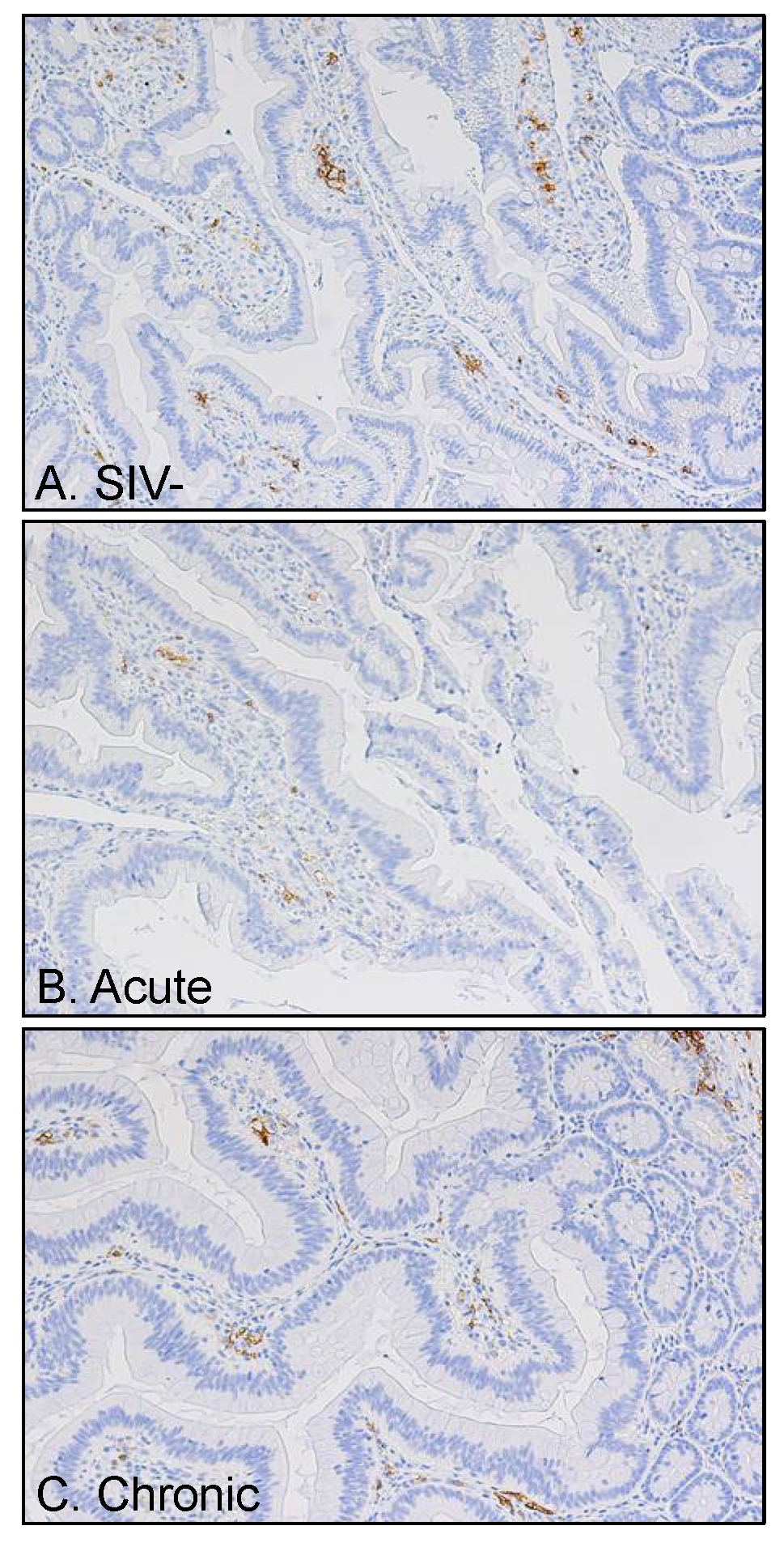

Supplement: Figure S6 — Immunohistochemical assessment of CD11c expression in the jejunum samples collected from SIVagmSab-infected PTMs. CD11c expression is decreased during acute infection (b) compared to preinfection (a) and chronic infection (c). Magnification: 10×. (TIFF) [file ppat.1003600.s006.tiff]

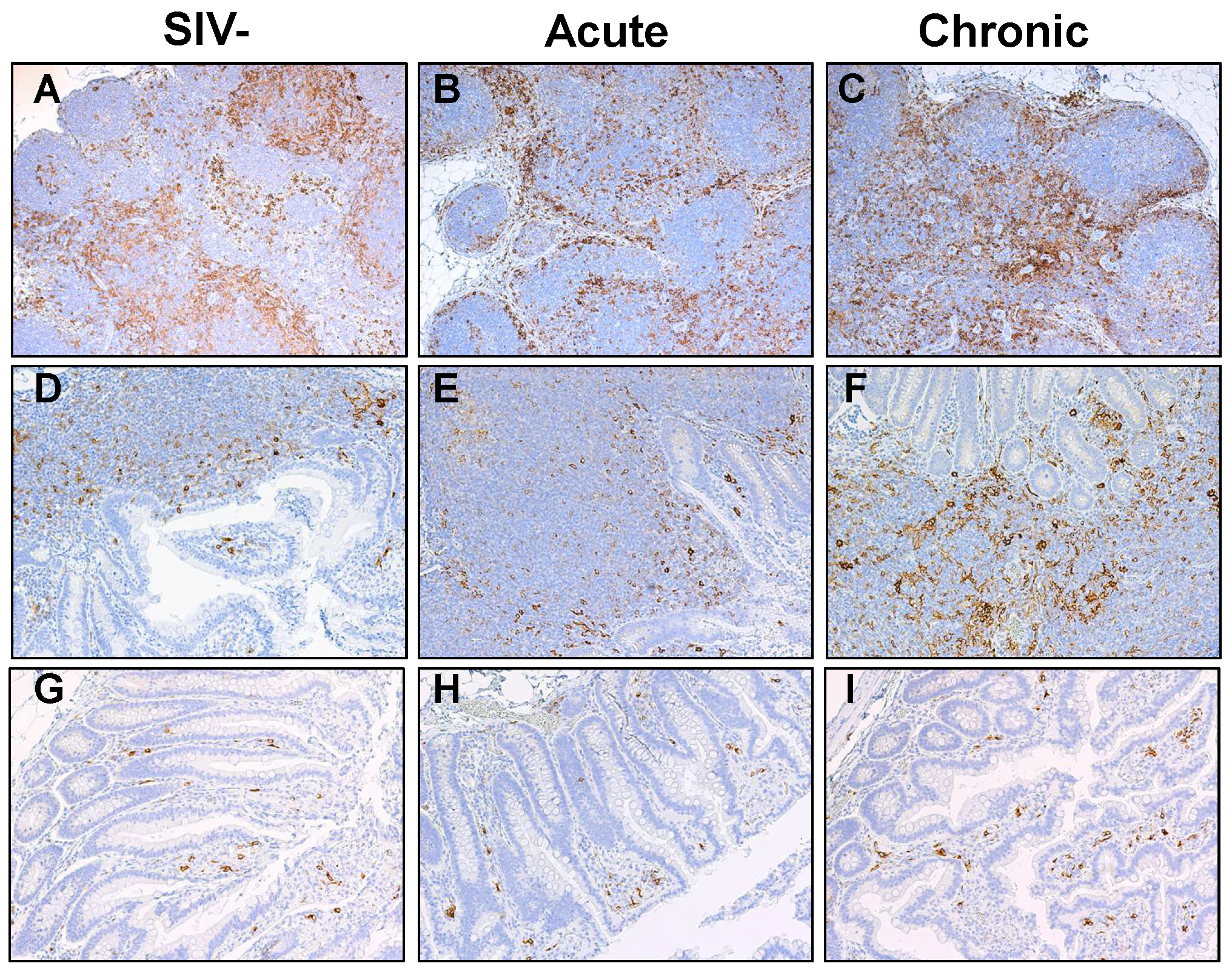

Supplement: Figure S7 — Immunohistochemical assessment of CD11c expression in tissues collected from SIVagmSab-infected AGMs. No difference in the expression of CD11c in the LNs collected prior to infection (a), as well as during acute (b) and chronic (c) SIVagmSab infection. CD11c expression in the Peyer's patches (f) and in the lamina propria (i) increased during the chronic stage of infection compared to preinfection levels (d and g) and those observed during the acute infection (e and h). Magnification: 10×. (TIFF) [file ppat.1003600.s007.tiff]

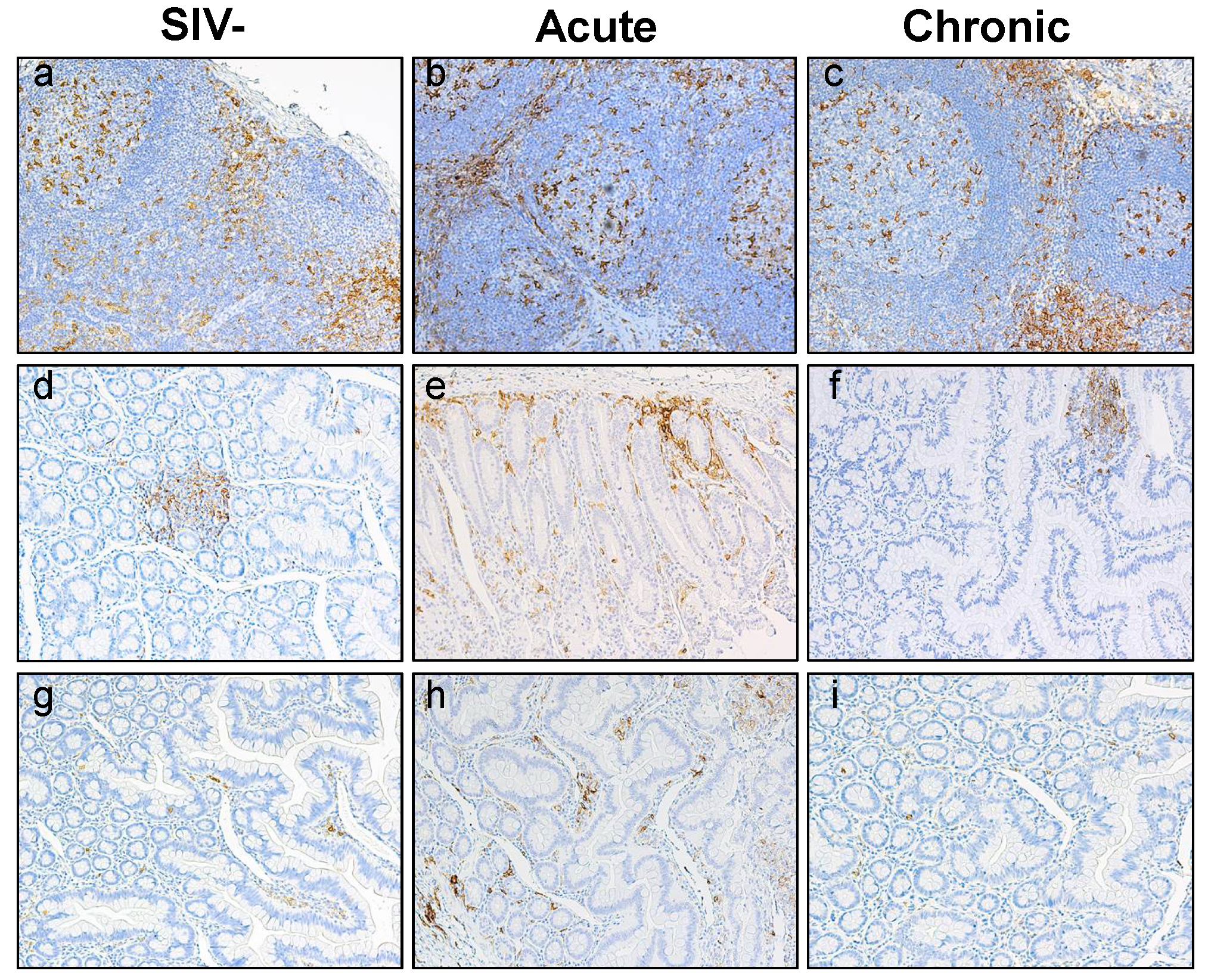

Supplement: Figure S8 — Immunohistochemical assessment of CD11c expression in tissues collected from SIVagmSab-infected RMs. No difference in the expression of CD11c in the LNs collected prior to infection (a), as well as during acute (b) and chronic (c) SIVagmSab infection. CD11c expression increased in the lamina propria during acute infection (e and h) compared to preinfection levels (d and g) and those observed during the chronic infection (f and i). The CD11c+ cells are mainly present in the lymphoid follicles (d–f). Magnification: 10×. (TIFF) [file ppat.1003600.s008.tiff]

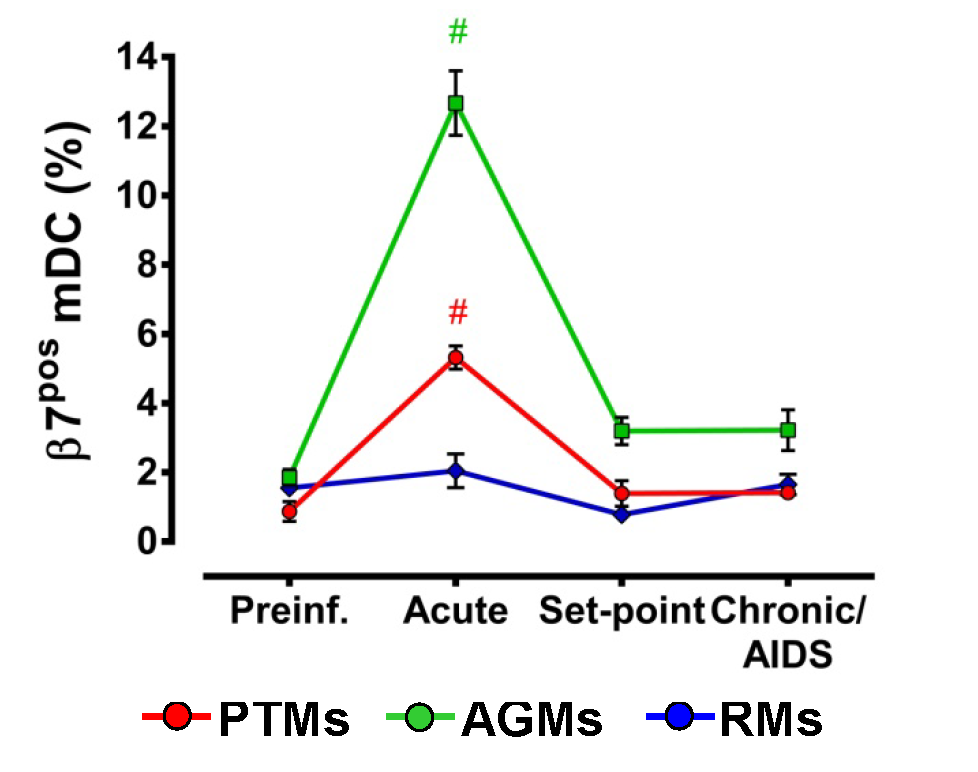

Supplement: Figure S9 — mDC mobilization to the intestine occurs in pathogenic (PTMs) SIVagm infection. Assessment of α4β7 expression on circulating mDCs in SIVagm-infected PTMs (red), AGMs (green) and RMs (blue). # signifies p<0.01 changes from the baseline levels within the same animal group (Anova). (TIF) [file ppat.1003600.s009.tif]
